# Supplementary material for: High yield electrosynthesis of oxygenates from CO using a relay Cu-Ag co-catalyst system
Source: Nat Commun. 2024 May 8;15:3892. doi: 10.1038/s41467-024-48083-w (PMC11078980; doi:10.1038/s41467-024-48083-w)
Supplement: Supplementary file 1 — Supplementary Information [file 41467_2024_48083_MOESM1_ESM.pdf]

**Supplementary Information**

# High yield electrosynthesis of oxygenates from CO using a relay Cu-Ag co-catalyst system

Nannan Meng[1,§], Zhitan Wu[1,2,§], Yanmei Huang[3,§], Jie Zhang[1], Maoxin Chen[1,2], Haibin Ma[1], Hongjiao Li[4,✉], Shibo Xi[5], Ming Lin[6], Wenya Wu[6], Shuhe Han[7], Yifu Yu[3], Quan-Hong Yang[2], Bin Zhang[3,✉], Kian Ping Loh[1,2,7,✉]

[1]Department of Chemistry, National University of Singapore, 3 Science Drive 3, Singapore 117543

[2]Joint School of National University of Singapore and Tianjin University, International Campus of Tianjin University, Binhai New City, Fuzhou 350207, China

[3]Institute of Molecular Plus, Department of Chemistry, Tianjin University, Tianjin 300072, China

[4]School of Chemical Engineering, Sichuan University, Chengdu, Sichuan 610065, China

[5]Institute of Chemical and Engineering Sciences, Agency of Science Technology and Research, 1 Pesek Road, Jurong Island, Singapore

[6]Institute of Materials Research and Engineering, Agency of Science Technology and Research, 2 Fusionopolis Way, #08-03, Innovis 138634, Singapore

[7]Department of Applied Physics, The Hong Kong Polytechnic University, Hung Hom, Kowloon, Hong Kong

[§]These authors contributed equally

*e-mail: hongjiao.li@scu.edu.cn (H. L.); bzhang@tju.edu.cn (B. Z.); chmlohkp@nus.edu.sg (K. L.)

# Contents

**Supplementary Figure 1.** Characterization of Ag and Cu.
**Supplementary Figure 2.** The linear sweep voltammetry measurement.
**Supplementary Figure 3.** The gas stripping measurement.
**Supplementary Figure 4.** The electric force field effect on CO adsorbate.
**Supplementary Figure 5.** The morphologies of Cu-Ag composites.
**Supplementary Figure 6.** The physical image for samples.
**Supplementary Figure 7.** The quantification of $C_1$ and $C_2$ gas products.
**Supplementary Figure 8.** Quantification of the acetate anion product.
**Supplementary Figure 9.** The quantification of volatile liquid products.
**Supplementary Figure 10.** The structure of the gas diffusion electrode (GDE).
**Supplementary Figure 11.** The performances of Cu and Cu-Ag composites for CO electroconversion.
**Supplementary Figure 12.** The typical *U-t* curves over Cu/30Ag under the different applied current densities.
**Supplementary Figure 13.** The performance comparison.
**Supplementary Figure 14.** Mass activities of Cu and Cu-Ag composites.
**Supplementary Figure 15.** Characterization of the used Cu and used Cu-Ag composites.
**Supplementary Figure 16.** The influence of CO flow rate on the performance.
**Supplementary Figure 17.** The performance comparison.
**Supplementary Figure 18.** The scheme structure of the membrane electrode assembly (MEA).
**Supplementary Figure 19.** Characterization of the Cu/30Ag composites used in the MEA cell.
**Supplementary Figure 20.** The *operando* X-ray absorption measurement.
**Supplementary Figure 21.** Atomic-level observation of the surface component of Cu/30Ag.
**Supplementary Figure 22.** Characterization of the physical mixture sample.
**Supplementary Figure 23.** The CO-EC performance of the physical mixture sample.
**Supplementary Figure 24.** Characterization of the interface-rich sample.
**Supplementary Figure 25.** The CO-EC performance of the interface-rich sample.
**Supplementary Figure 26.** The cyclic voltammetry measurement.
**Supplementary Figure 27.** Double-layer capacitance measurements of Cu, Ag, Cu-Ag composites and GDE.
**Supplementary Figure 28.** Roughness factors (RF) of the different samples.
**Supplementary Figure 29.** The *in situ* Raman spectra in Ar for comparison.
**Supplementary Figure 30.** The Gibbs free energy change of CO hydrogenation with the change of the applied potentials on Ag(100).
**Supplementary Figure 31.** The legend for the video.
**Supplementary Figure 32.** The typical snapshots of *HCO transfer of crossing the boundary of Ag(111) and Cu(111) with the boundary density of 1/9.

**Supplementary Figure 33.** The trajectory profile of *HCO from Ag(111) to Cu(111) and the corresponding energy change.

**Supplementary Figure 34.** The interface structure of Cu(100)|Ag(100)

**Supplementary Table 1**. The CO-EC performance of Cu at different applied current densities (unit: % for FE, mA $cm^{-2}$ for *j*).

**Supplementary Table 2**. The CO-EC performance of Cu/10Ag at different applied current densities (unit: % for FE, mA $cm^{-2}$ for *j*).

**Supplementary Table 3**. The CO-EC performance of Cu/30Ag at different applied current densities (unit: % for FE, mA $cm^{-2}$ for *j*).

**Supplementary Table 4**. The CO-EC performance of Cu/50Ag at different applied current densities (unit: % for FE, mA $cm^{-2}$ for *j*).

**Supplementary Table 5**. The CO-EC performance of Cu/90Ag at different applied current densities (unit: % for FE, mA $cm^{-2}$ for *j*).

**Supplementary Table 6**. The CO-EC performance of Ag for oxygenates generation at 800 mA $cm^{-2}$ (unit: % for FE, mA $cm^{-2}$ for *j*).

**Supplementary Table 7**. The CO-EC performance of Cu/30Ag at 800 mA $cm^{-2}$ under a CO flow rate of 5 SCCM (unit: % for FE, mA $cm^{-2}$ for *j*).

**Supplementary Table 8**. The CO-EC performance of Cu/30Ag at 1000 mA $cm^{-2}$ electrolysis with different flow rates of electrolyte (unit: % for FE, mA $cm^{-2}$ for *j*).

**Supplementary Table 9**. The CO-EC performance of Cu/30Ag at 1200, and 1400 mA $cm^{-2}$ electrolysis with a flow rate of 100 mL $min^{-1}$ electrolyte (unit: % for FE, mA $cm^{-2}$ for *j*).

**Supplementary Table 10**. The performance list of typical catalysts used for CO-to-oxygenate electroconversion at their respective optimized FE points and ambient conditions.

**Supplementary Table 11**. The performance list of Cu/30Ag used for CO-to-oxygenate electroconversion in MEA (unit: % for FE, mA $cm^{-2}$ for *j*).

**Supplementary Table 12**. The CO-EC performance of the physical mixture sample at different applied current densities (unit: % for FE, mA $cm^{-2}$ for *j*).

**Supplementary Table 13**. The CO-EC performance of the interface-rich sample at different applied current densities (unit: % for FE, mA $cm^{-2}$ for *j*).

**Supplementary Table 14**. The Gibbs free energy change of the CO hydrogenation and CO dimerization reactions on different surfaces. The reference moleculars are CO and $H_2$ in the vacuum.

**Supplementary Note 1.** A techno-economic analysis.

**Supplementary References (1-4)**

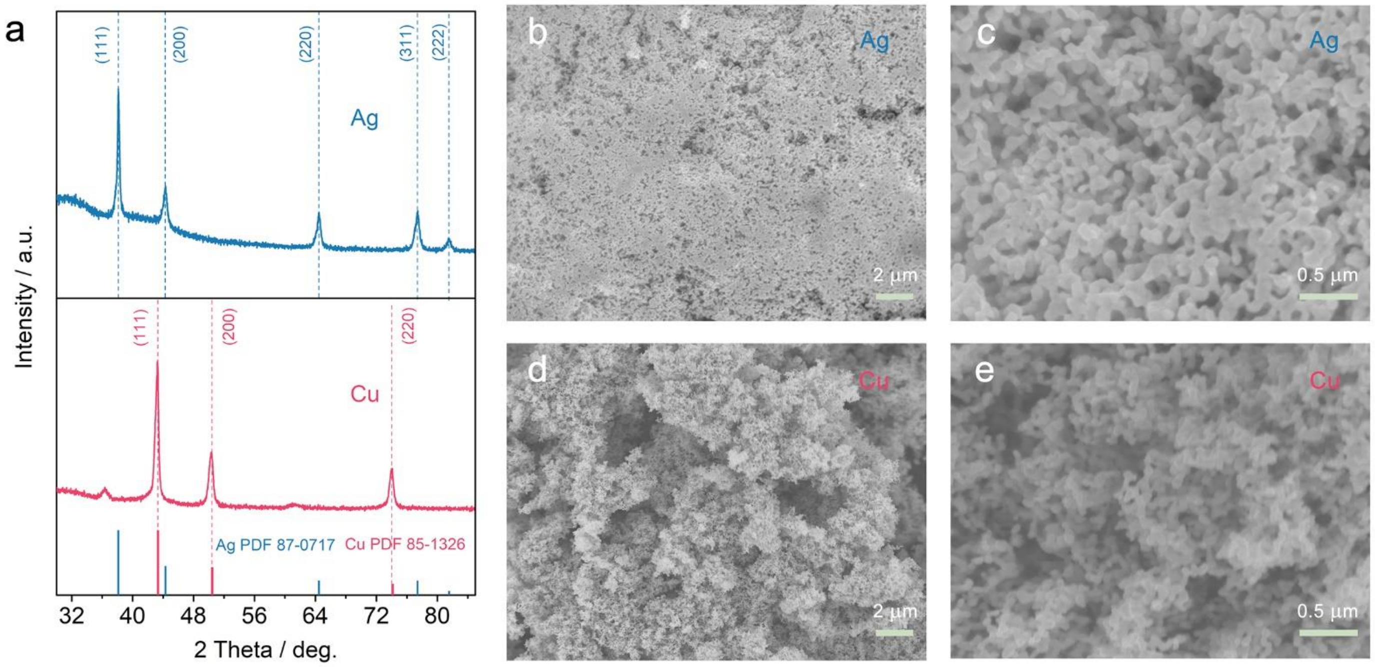


Supplementary Figure 1 │Characterization of Ag and Cu. (a) X-ray diffraction (XRD) patterns of Ag and Cu. Scanning electron microscope (SEM) images of Ag (b, c) and Cu (d, e) under different magnifications.

Cu and Ag are synthesized by liquid phase reduction in ammonia (see the Methods section in the text). As seen from Supplementary Fig. 1 a, the XRD pattern proves that Ag (PDF # 87-0717) is pure. For the Cu sample, metallic Cu is dominant (PDF # 85-1326), and some peaks at ~29$^o$ and 61$^o$ belonging to $Cu_2O$ (PDF # 78-2076) are observed due to partial surface oxidation in air. SEM images describe the branch-like morphology of Ag (Supplementary Figs. 1b,c) and nanoparticle shape of Cu (Supplementary Figs. 1d,e).

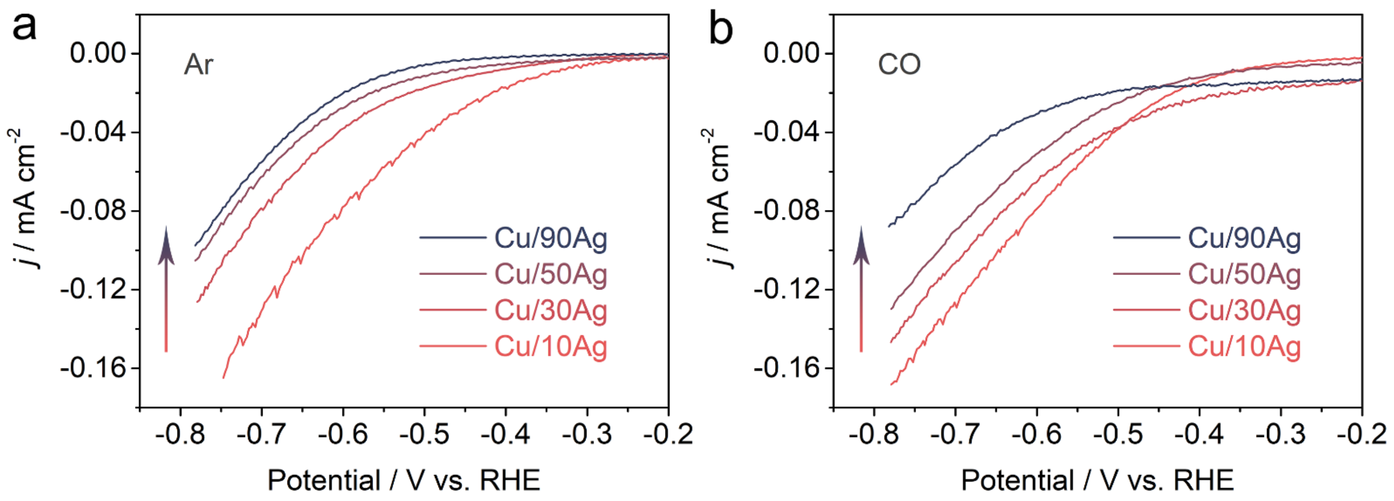


Supplementary Figure 2 ｜The linear sweep voltammetry measurement. The responses of linear sweep voltammetry curves to Ag content in Cu-Ag composites in Ar (a) and CO (b) atmospheres.

With increasing Ag content in Cu-Ag composites, HER activity of Cu can be efficiently suppressed (Supplementary Fig. 2a) and the reaction kinetics of CO electroreduction can be also steered (Supplementary Fig. 2b).

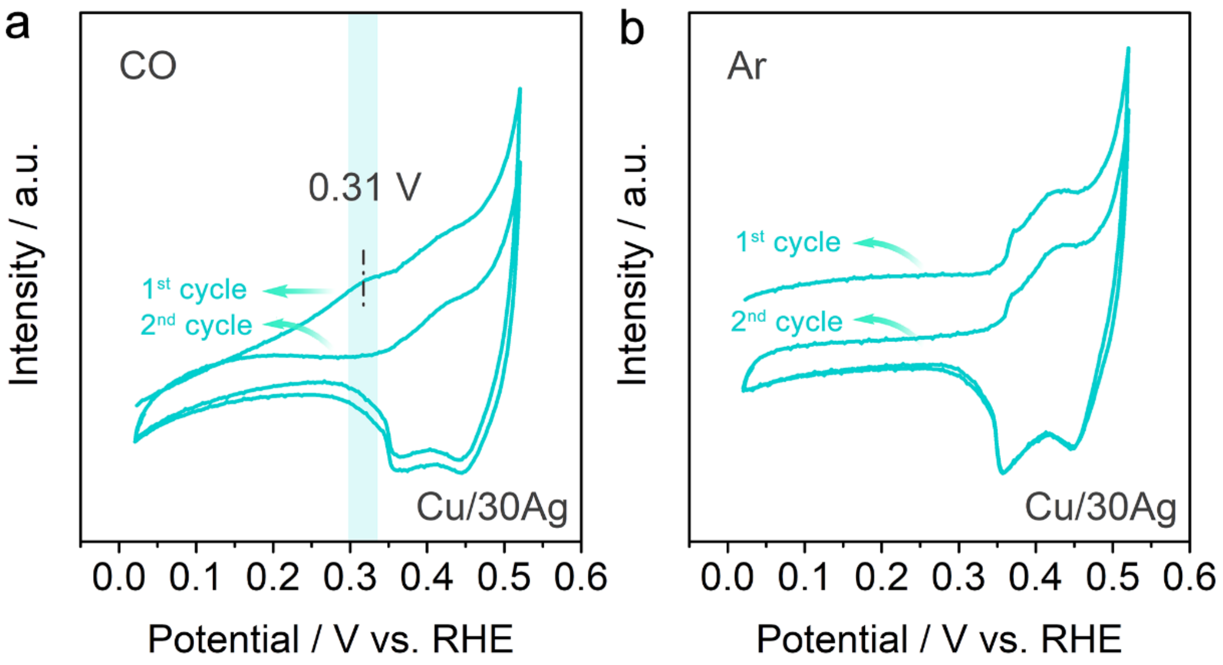


Supplementary Figure 3 | The gas stripping measurement. The stripping curve over Cu/30Ag in CO (a) and Ar (b) atmospheres.

Compared with the CO stripping peak at 0.27 V over Cu, the more positive peak position at 0.31 V over Cu/30Ag, indicating Ag can efficiently enhance the CO capture in the solution.

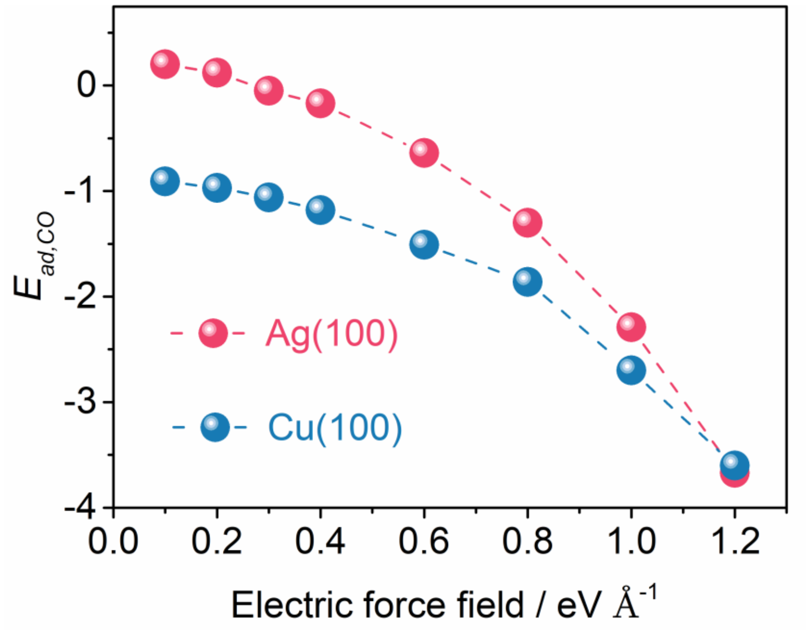


Supplementary Figure 4 | The electric force field effect on CO adsorbate.

The results depicted in Supplementary Fig. 4 substantiate the assertion that CO adsorption is more robust on Ag than on Cu, particularly with an applied electric force field exceeding ~1.2 eV/Å.

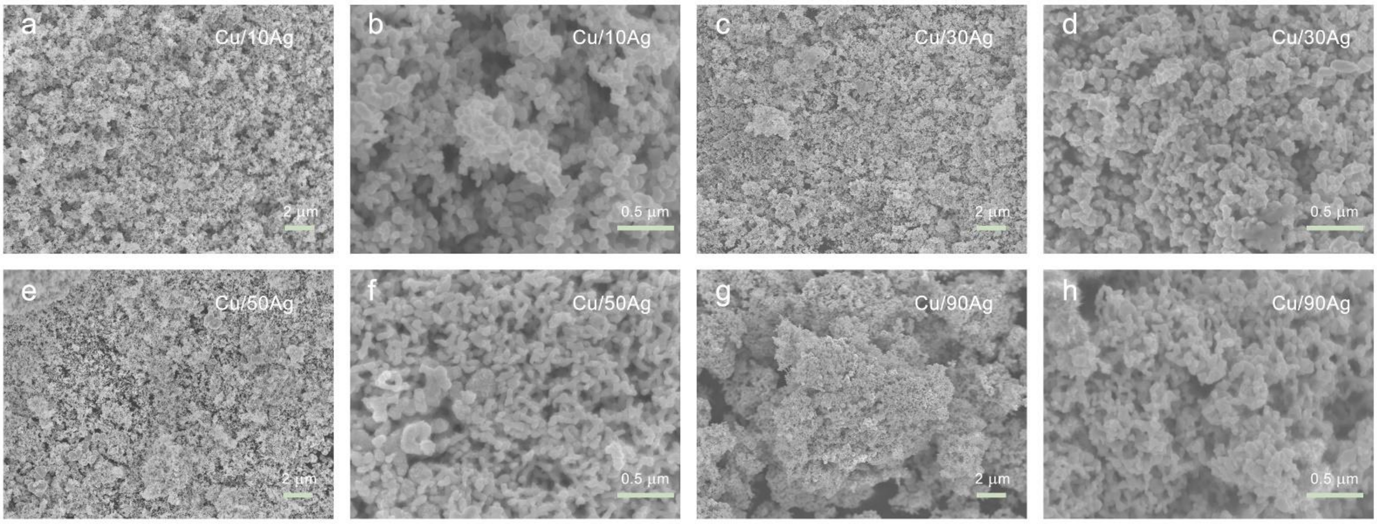


Supplementary Figure 5 │The morphologies of Cu-Ag composites. SEM images of Cu/10Ag (a, b), Cu/30Ag (c, d), Cu/50Ag (e, f), and Cu/90Ag (g, h).

With increasing Ag content in the composites, the morphology changes from a nanoparticle shape to a branch-like shape.

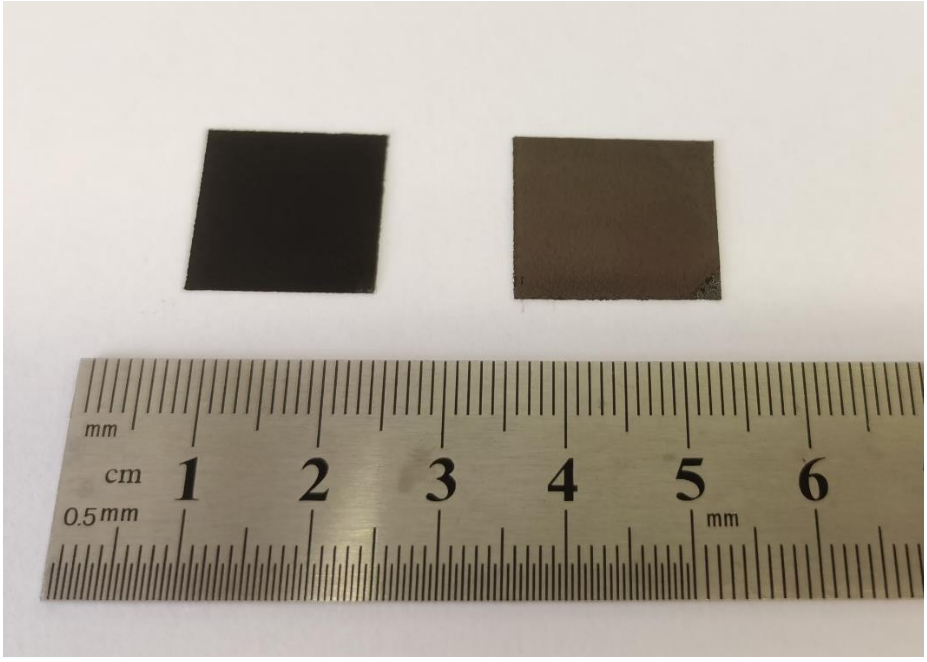


Supplementary Figure 6 │The physical image for samples. Photographs of the Cu-covered gas diffusion electrode (left) and Cu/30Ag-covered gas diffusion electrode (right).

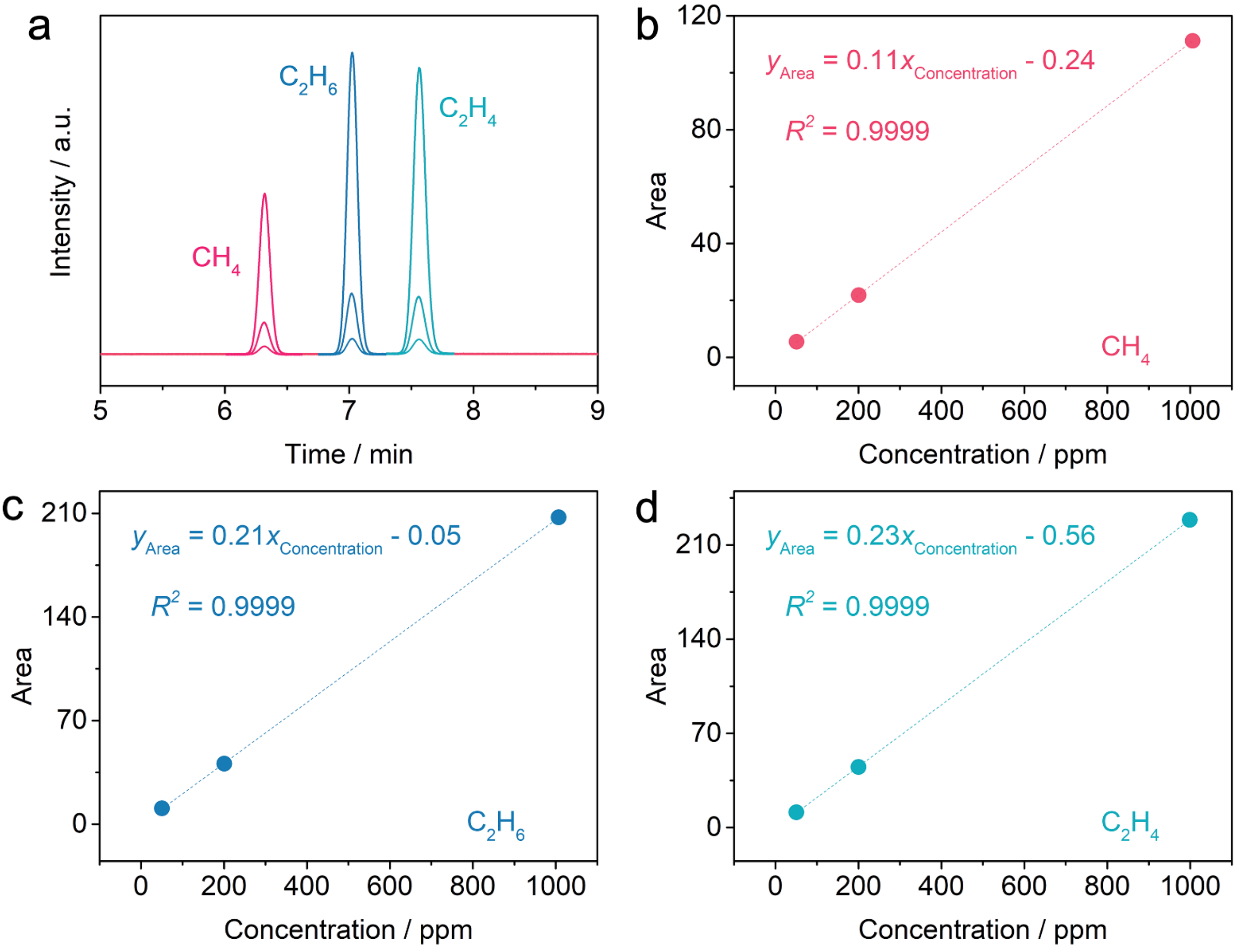


Supplementary Figure 7 | The quantification of $C_1$ and $C_2$ gas products. (a) Gas chromatograph (GC) chromatograms of $CH_4$, $C_2H_6$, and $C_2H_4$. (b-d) The standard curves of $CH_4$, $C_2H_6$, and $C_2H_4$.

C1 and C2 gas products were quantified using GC. As seen from Supplementary Fig. 7a, the peaks of $CH_4$, $C_2H_6$, and $C_2H_4$ appear in sequential order and are well separated. The standard curves for $CH_4$, $C_2H_6$, and $C_2H_4$ (Supplementary Figs. 7b-d) are $y_{(\text{Area})} = 0.11x_{(\text{Concentration, CH4})} - 0.24$, $y_{(\text{Area})} = 0.21x_{(\text{Concentration, C2H6})} - 0.05$, and $y_{(\text{Area})} = 0.23x_{(\text{Concentration, C2H4})} - 0.56$, respectively. All correlation coefficients ($R^2$) are 0.9999, suggesting a good linear relationship between the GC area and the corresponding concentration.

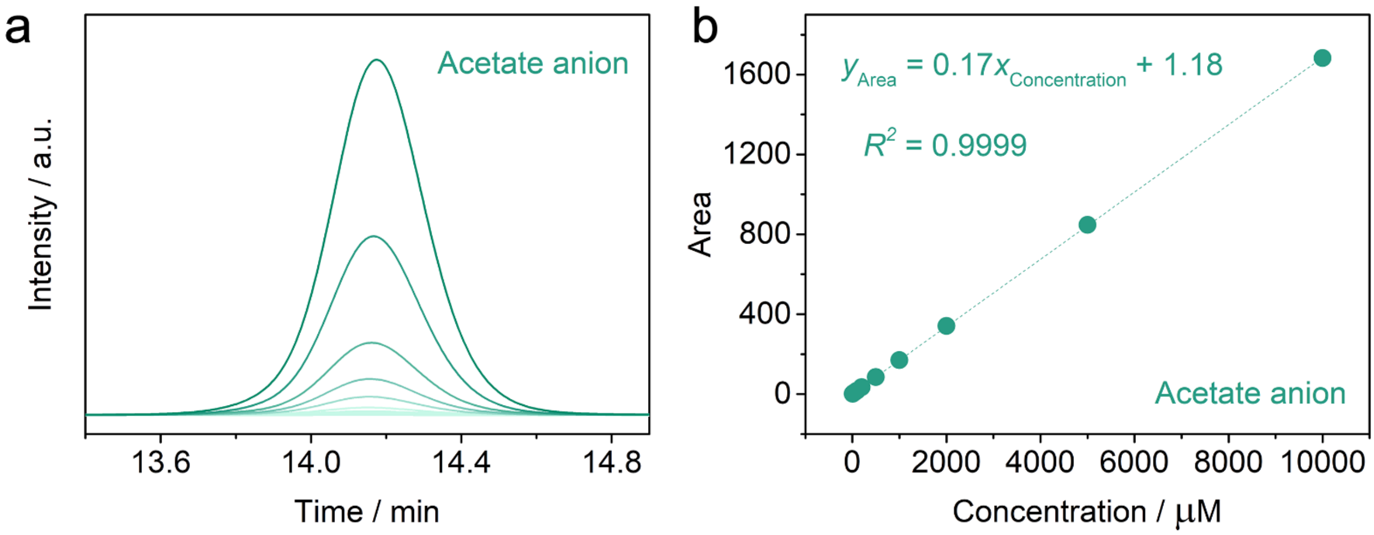


Supplementary Figure 8 │Quantification of the acetate anion product. (a) Liquid chromatograph (LC) chromatograms of acetate anion at different concentrations. (b) The standard curves of acetate anion.

Acetate was quantified using LC. As seen from Supplementary Fig. 8a, the acetate peaks show a good symmetrical peak shape. The standard curve for acetate (Supplementary Fig. 8b) is $y_{(\mathrm{Area})} = 0.17x_{(\mathrm{Concentration,\ Acetate\ anion})} + 1.18$. The correlation coefficient ($R^2$) is 0.9999, suggesting a good linear relationship between its LC area and the corresponding concentration.

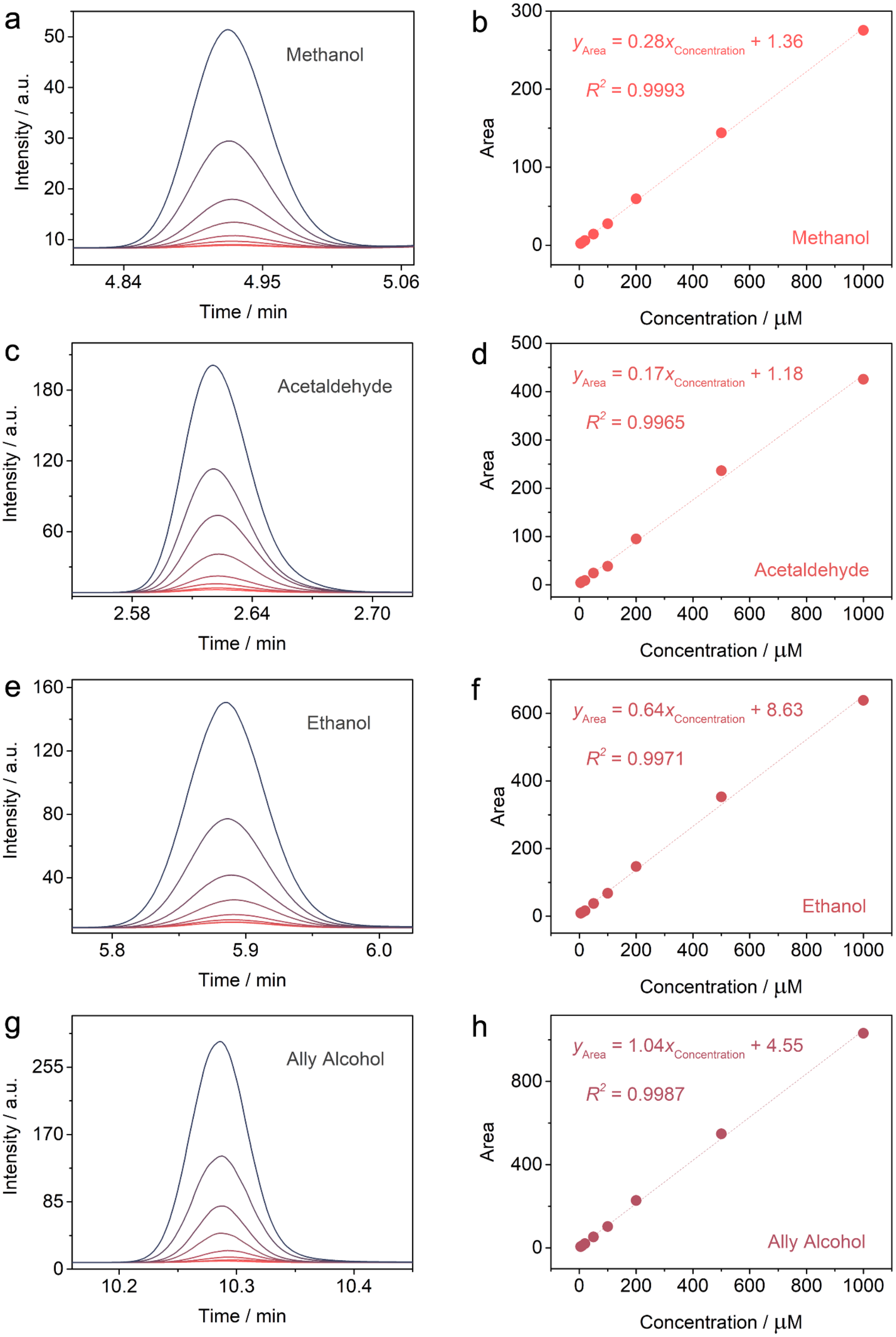


Supplementary Figure 9 │The quantification of volatile liquid products.

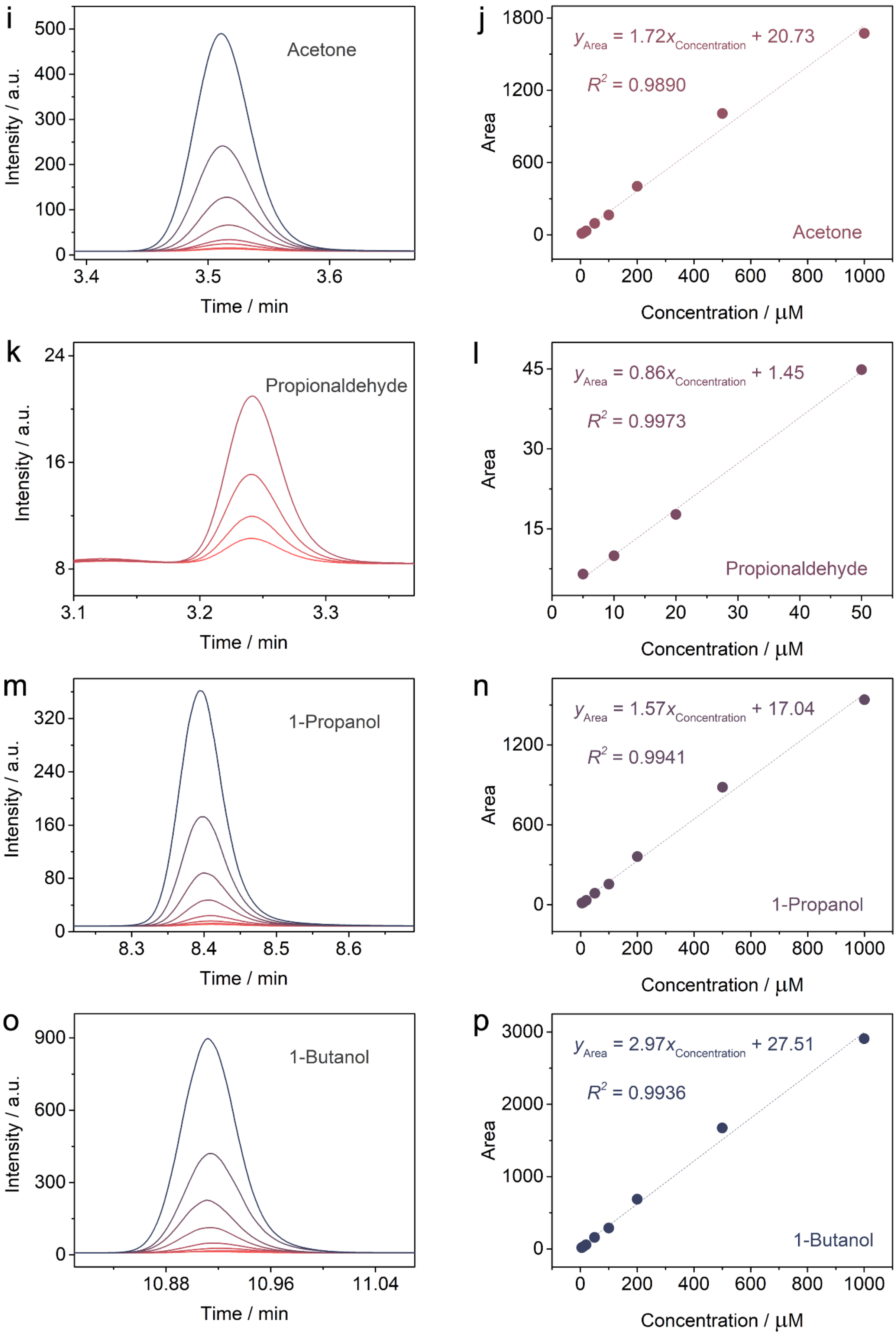


Supplementary Figure 9 | The quantification of volatile liquid products (continued). (a) Head space gas chromatograph (HSGC) chromatograms and the corresponding standard curves of methanol (a, b), acetaldehyde (c, d), ethanol (e, f), ally alcohol (g, h), acetone (i, j), propionaldehyde (k, l), 1-propanol (m, n), and 1-butanol (o, p).

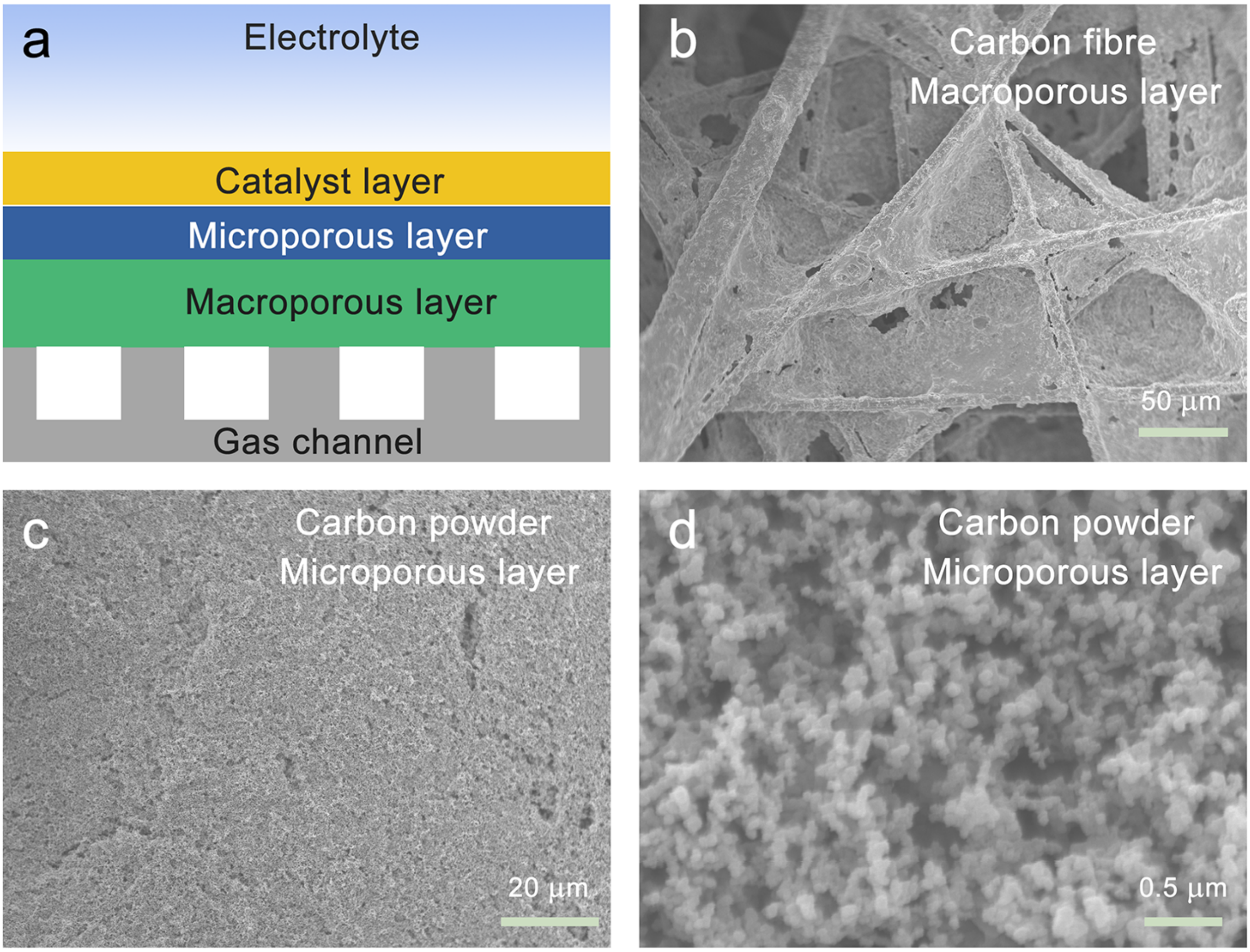


Supplementary Figure 10 | The structure of the gas diffusion electrode (GDE). (a) Scheme of the GDE structure. (b) SEM image of the macroporous structure. (c,d) SEM images of the microporous structure.

GDE is composed of a macroporous structure (green color) and microporous structure (indigo blue color) (Supplementary Fig. 10a). The microporous structure is composed of carbon fibre (Supplementary Fig. 10b), which tightly attaches to the gas chamber and facilitates gas penetration. The microporous structure is composed of carbon powder (Supplementary Figs. 10c, d), which is covered by a catalyst layer (yellow color in Supplementary Fig. 10a) and plays dual roles of support and gas penetration.

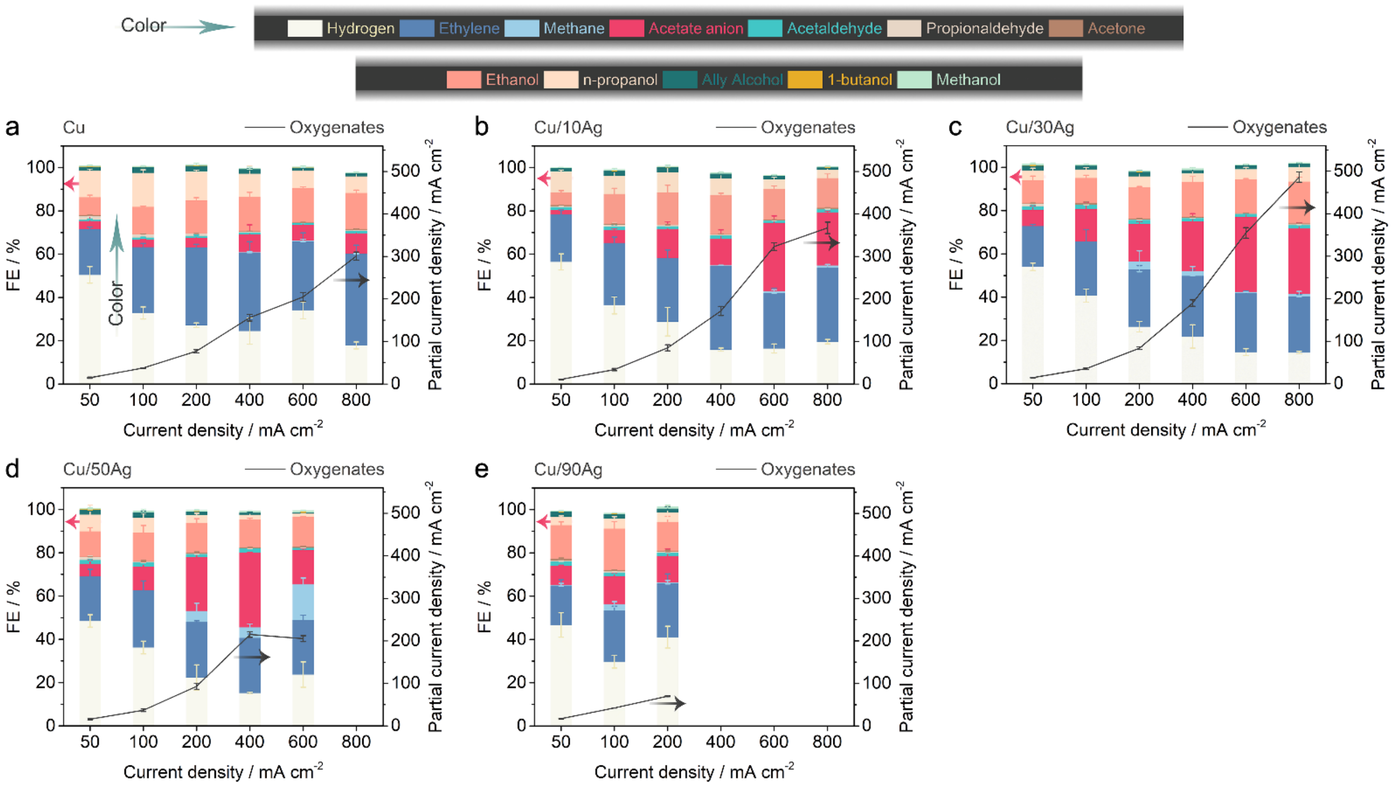


Supplementary Figure 11 | The performances of Cu and Cu-Ag composites for CO electroconversion. Error bars correspond to the Standard Deviation (SD) of three independent measurements.

The detailed product distributions of Cu and Cu-Ag composites are shown in Supplementary Fig. 11. The main oxygenated CO-EC products of Cu-Ag composites are ethanol and acetate anion.

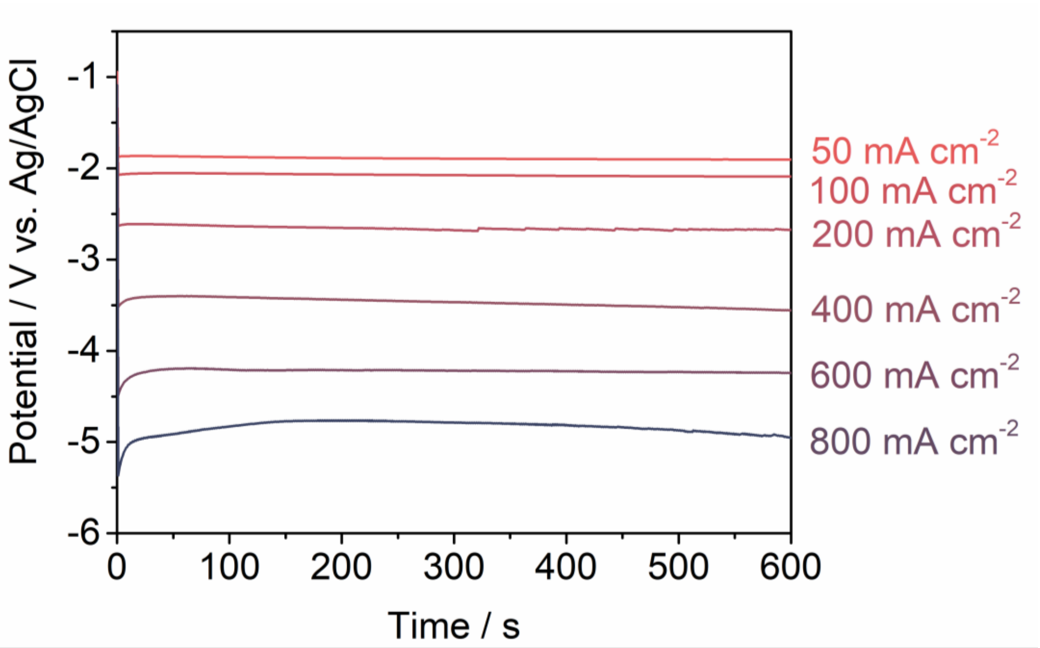


Supplementary Figure 12 | The typical *U*-*t* curves over Cu/30Ag under the different applied current densities.

The curves of *U*-*t* under the different applied current densities from 50 to 800 mA $cm^{-2}$ show a stable state, indicating the excellent catalytic stability of Cu/30Ag.

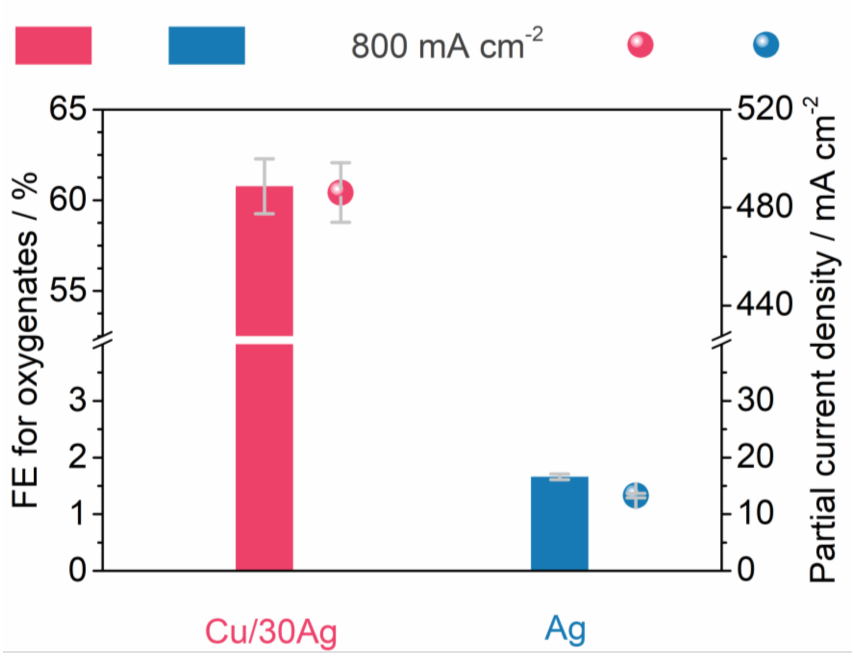


Supplementary Figure 13 | The performance comparison. The performance of oxygenates electrosynthesis over Cu/30Ag and Ag at the applied current density of 800 mA $cm^{-2}$. Error bars correspond to the Standard Deviation (SD) of three independent measurements.

The very weak performance for oxygenates electrosynthesis over pure Ag indicates the primary reaction zone for oxygenates generation is located on Cu.

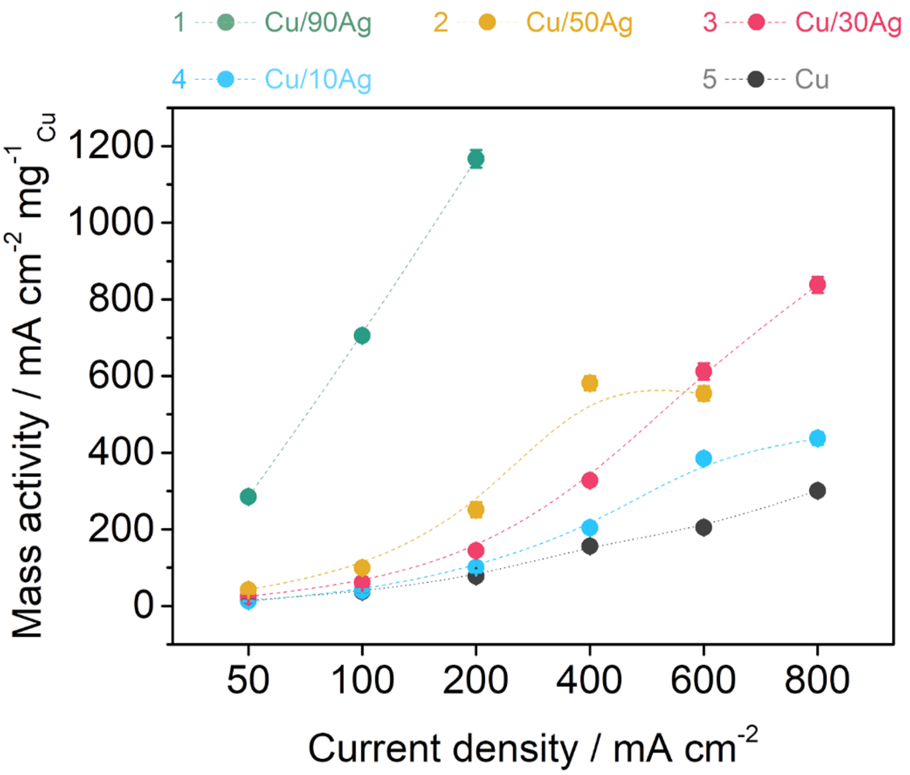


Supplementary Figure 14 │Mass activities of Cu and Cu-Ag composites. Error bars correspond to the Standard Deviation (SD) of three independent measurements.

The detailed product distributions of the Cu and Cu-Ag composites are shown in Supplementary Fig. 14. The main oxygenated mass activity of all composites significantly increases with increasing Ag-to-Cu ratio, further proving the Ag-assisted performance enhancement.

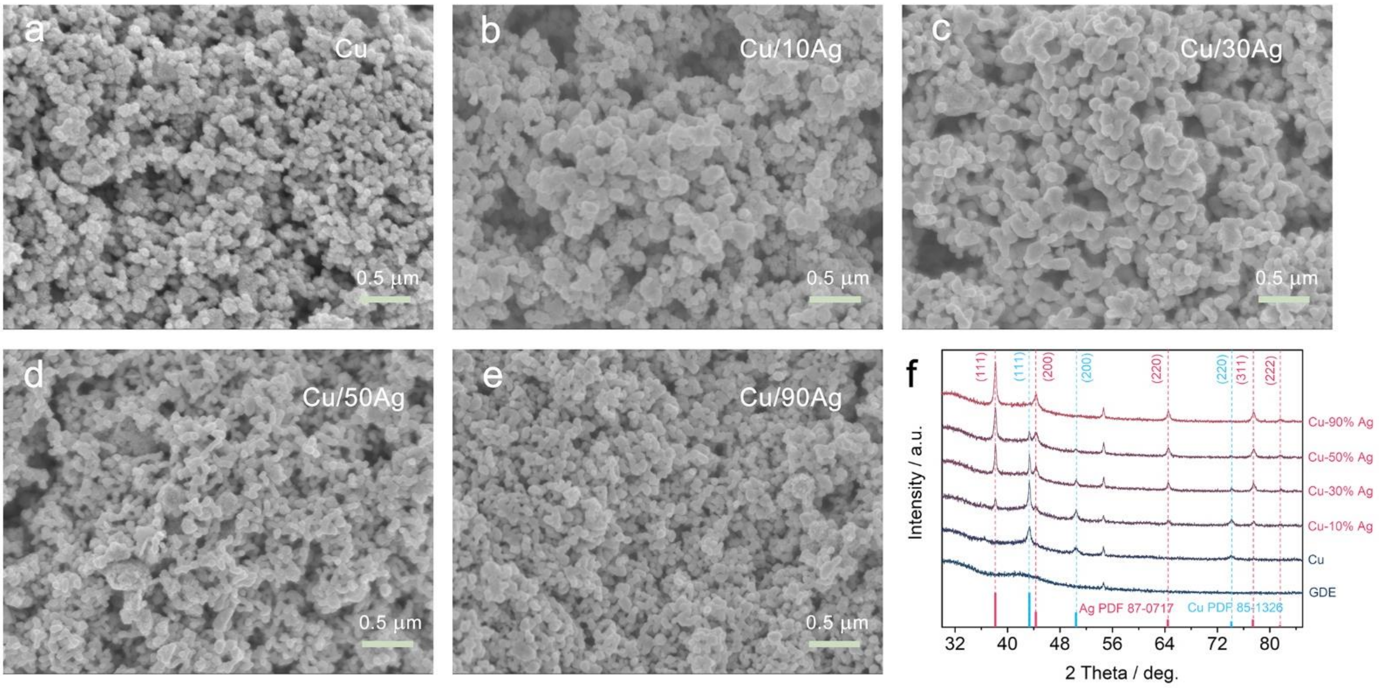


Supplementary Figure 15 │Characterization of the used Cu and used Cu-Ag composites. SEM images of Cu (a), Cu/10Ag (b), Cu/30Ag (c), Cu/50Ag (d) and Cu/90Ag (e). (f) XRD patterns of GDE, Cu and Cu-Ag composites.

After CO-EC in the flow cell, the Cu and Cu-Ag composites maintained their morphologies (Supplementary Figs. 15a-e) and phase structures (Supplementary Fig. 15f).

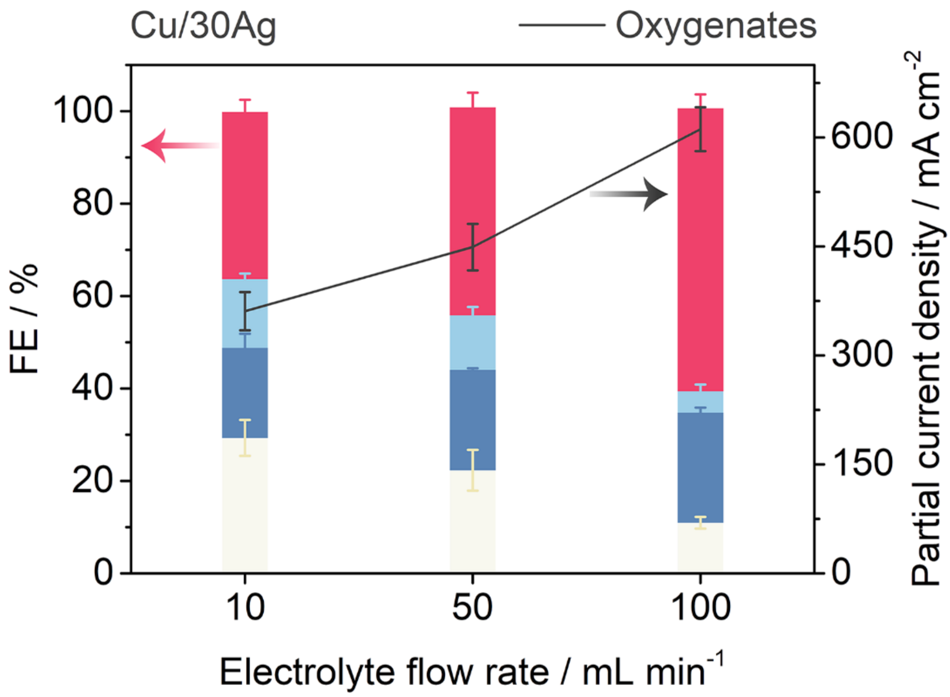


Supplementary Figure 16 │The influence of CO flow rate on the performance. Cu/30Ag CORR performances under 10 mL min$^{-1}$, 50 mL min$^{-1}$ and 100 mL min$^{-1}$ flow rates of the cathode electrolyte. Error bars correspond to the Standard Deviation (SD) of three independent measurements.

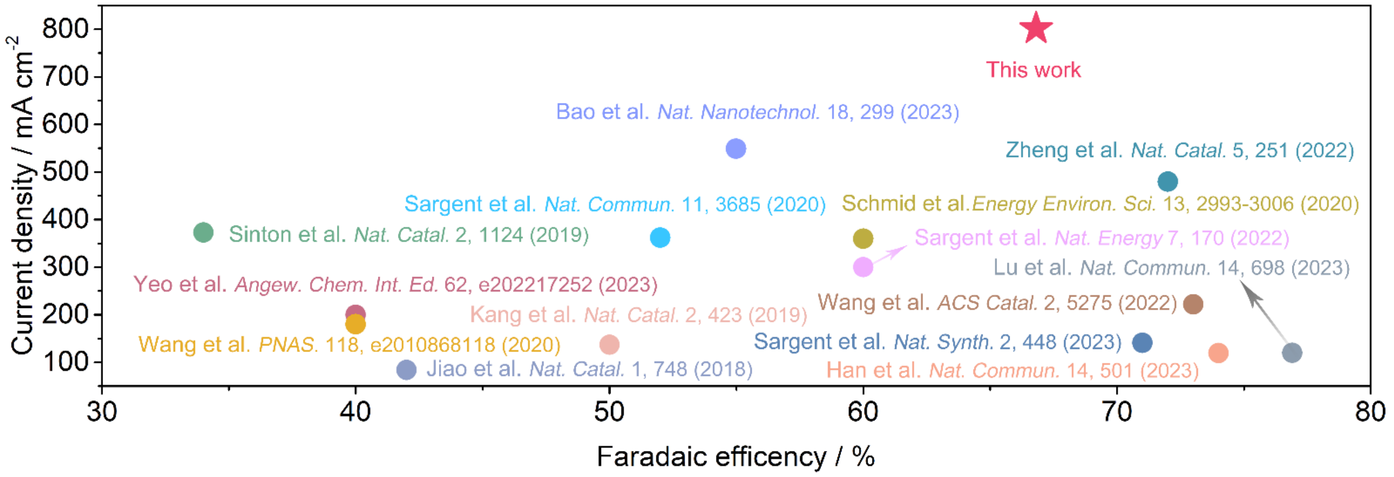


Supplementary Figure 17 | The performance comparison. Current density and Faradaic efficiency for CO-to-oxygenates electrosynthesis over Cu/30Ag and other works at ambient conditions (25 ºC, 1 atm).

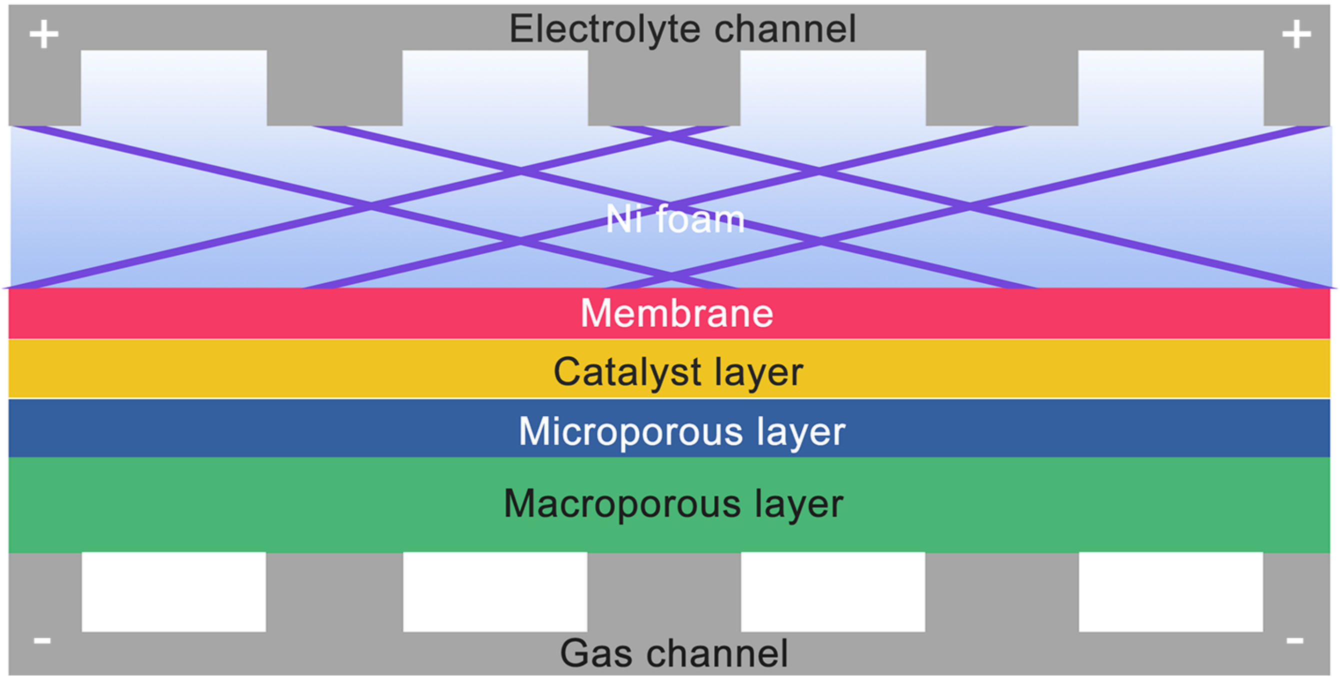


Supplementary Figure 18 | The scheme structure of the membrane electrode assembly (MEA).

The MEA is composed of a GDE, an anion exchange membrane (AEM) and Ni foam. Electrolyte only flows into the anode side though porous Ni foam. During the CO-EC reaction, most of the products cross the AEM into the anodic electrolyte, and a small fraction of the products will be blown into the downstream trap.

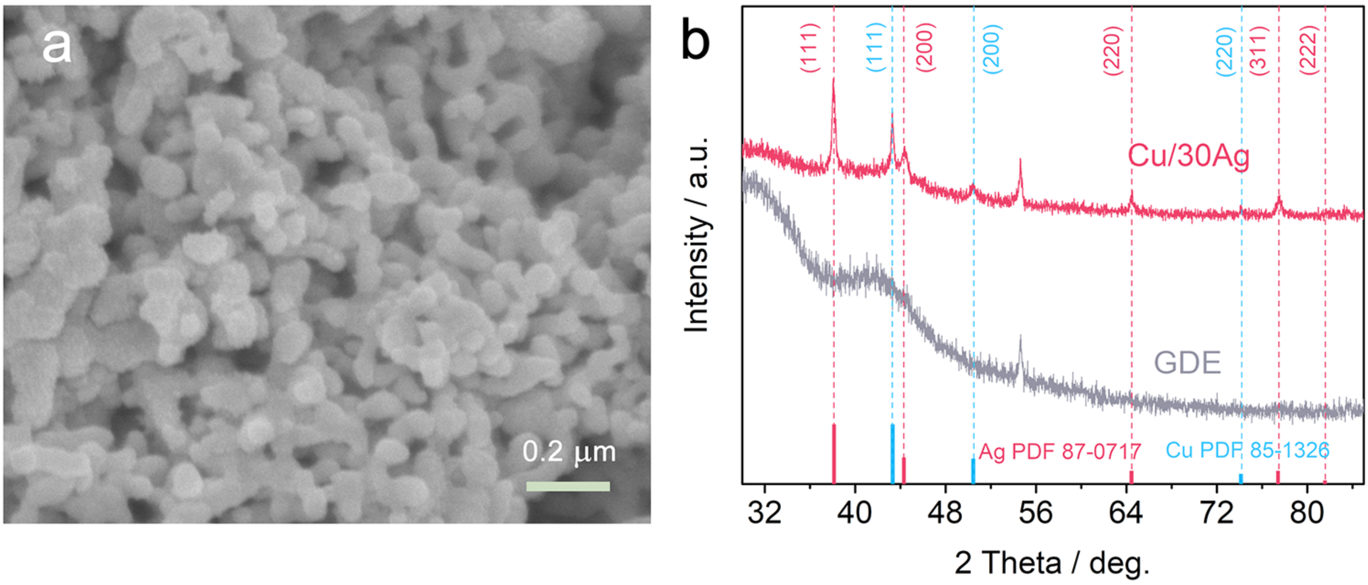


Supplementary Figure 19 | Characterization of the Cu/30Ag composites used in the MEA cell. SEM image and XRD pattern of used Cu/30Ag in the MEA cell.

After CO-EC in the MEA cell, the Cu/30Ag composite well maintains its morphology (Supplementary Fig. 19a) and phase structure (Supplementary Fig. 19b).

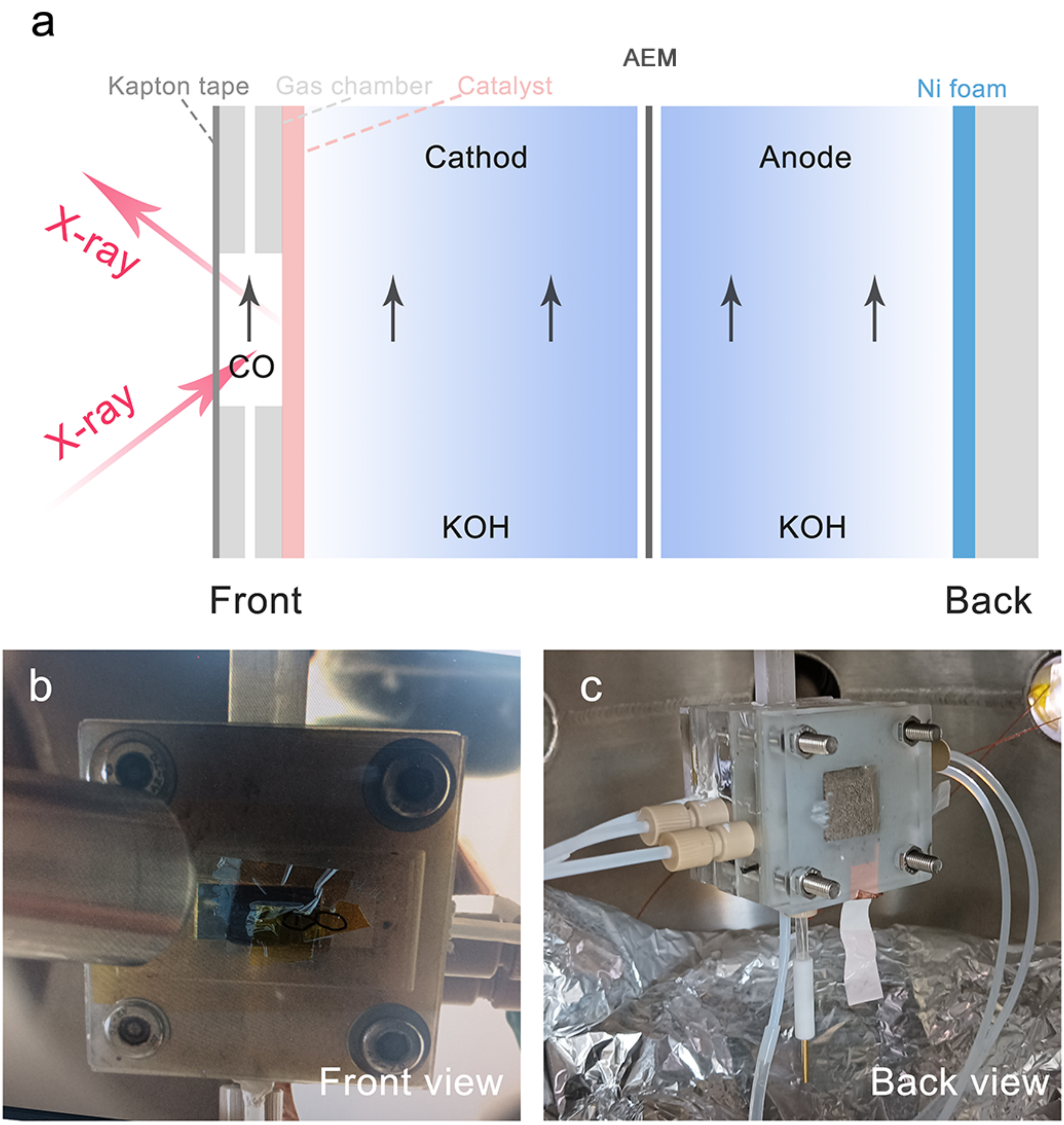


Supplementary Figure 20 │The *operando* X-ray absorption measurement. The scheme structure of *operando* X-ray absorption testing equipment (a) and the corresponding flow cell (b, c).

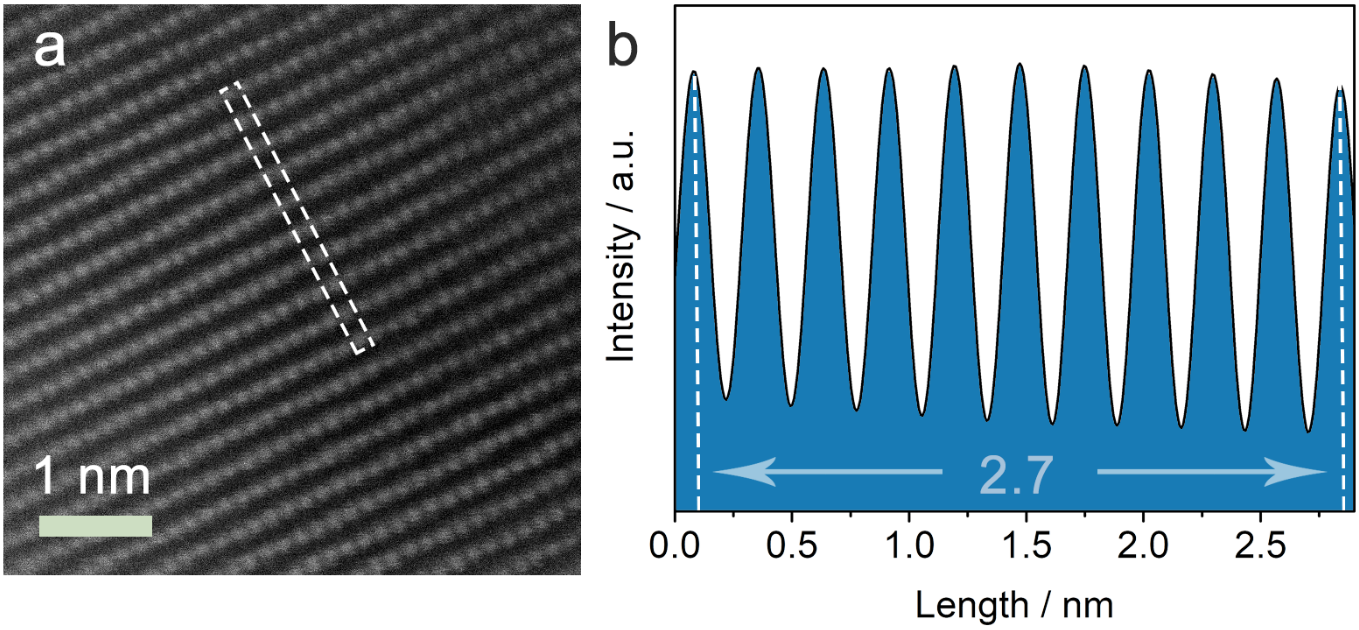


Supplementary Figure 21 │Atomic-level observation of the surface component of Cu/30Ag. (a) Scanning transmission electron microscopy in the high-angle annular dark-field mode (STEM) and (b) the lattice space measurement for the white rectangle in (a).

STEM and lattice space measurements prove that the surface of Cu appears in the form of CuO (0.27 nm corresponds to the (110) crystal plane of CuO).

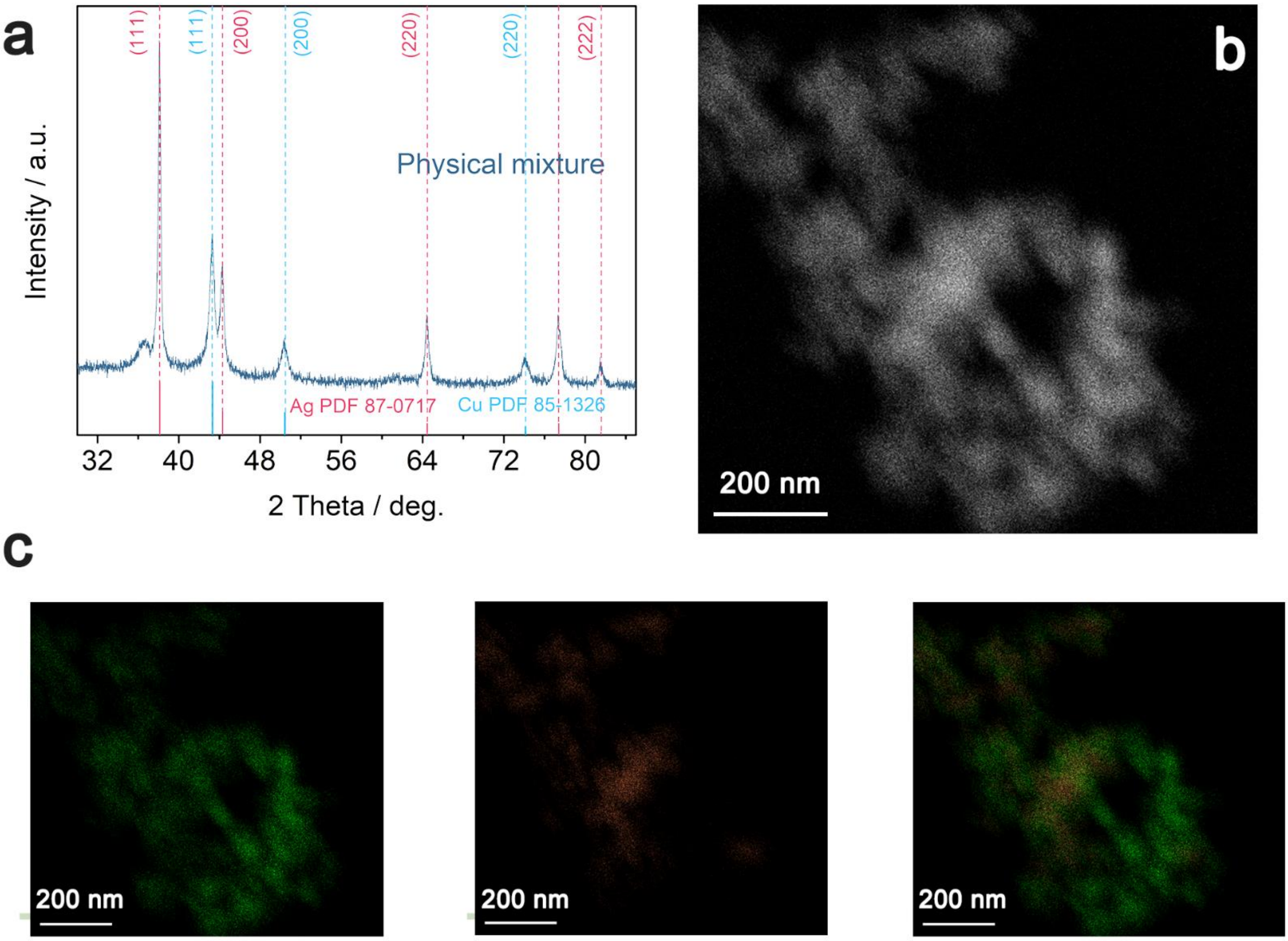


Supplementary Figure 22 | Characterization of the physical mixture sample. (a) XRD, (b) transmission electron microscope image (TEM) and (c) the corresponding energy dispersive spectra (c) of the physical mixture sample.

Supplementary Fig. 22a shows that the physical mixture sample is composed of Cu metal (PDF # 85-1326) and Ag metal (PDF # 87-0717). TEM and the corresponding energy dispersive spectra (Supplementary Figs. 22b, c) prove that Cu and Ag are well mixed together.

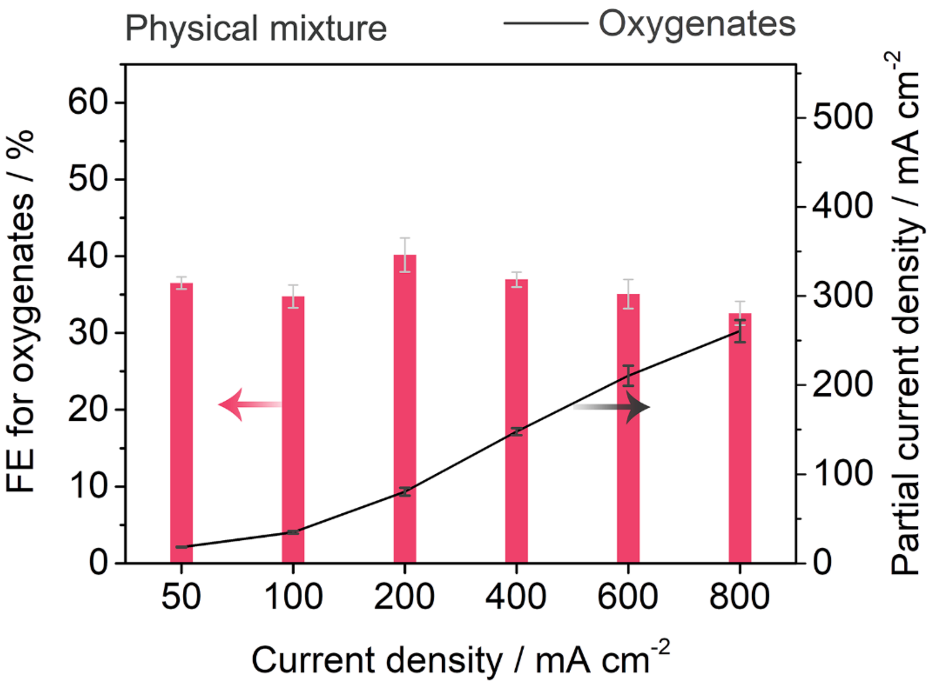


Supplementary Figure 23 │The CO-EC performance of the physical mixture sample. Current density-dependent Faradaic efficiency and partial current densities for oxygenates of the physical mixture sample for CO-EC. Error bars correspond to the Standard Deviation (SD) of three independent measurements.

For the physical mixture, its performance is similar as that of neat Cu, suggesting that a decent distance between Cu and Ag is an essential condition for performance enhancement.

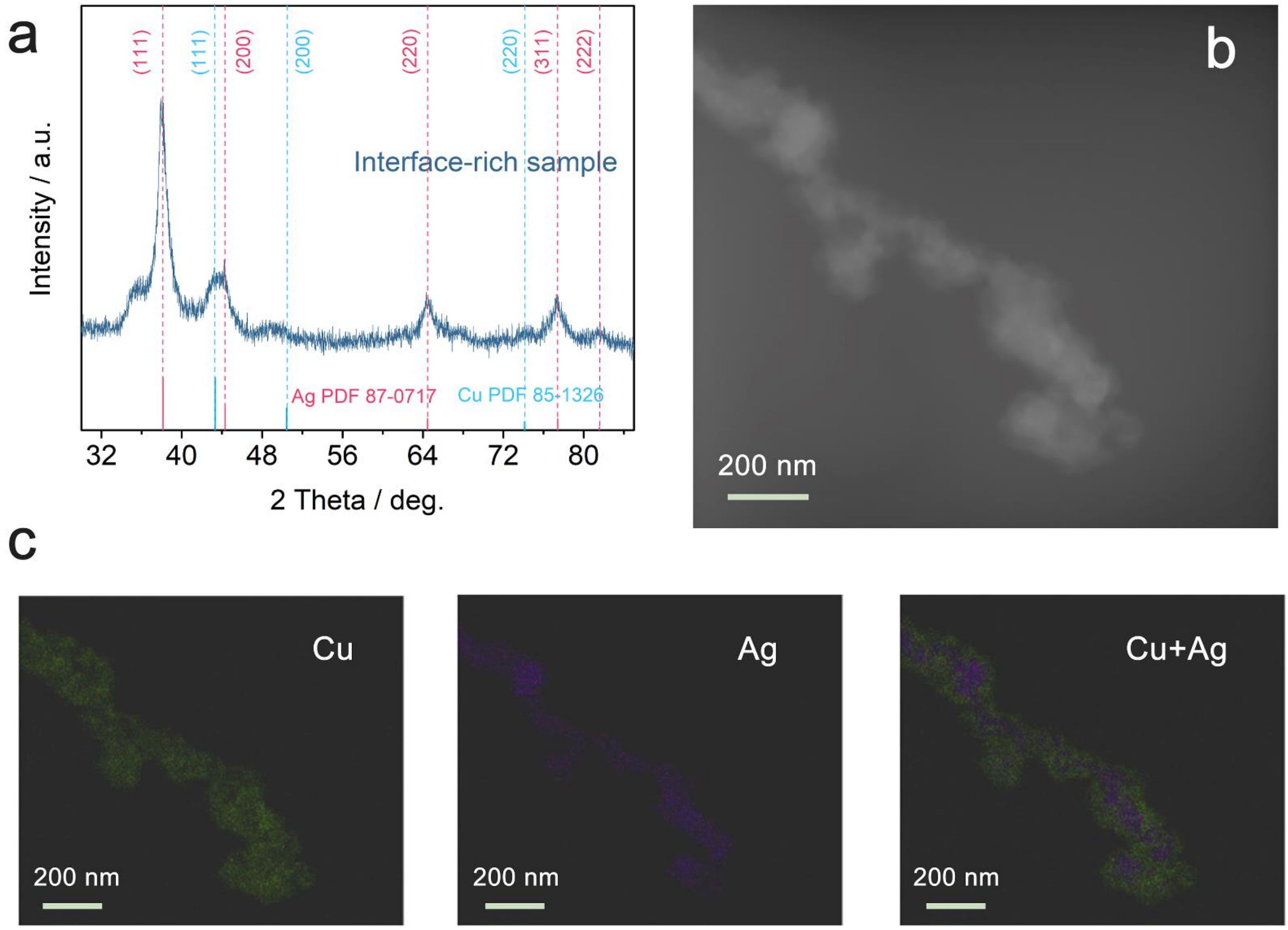


Supplementary Figure 24 │Characterization of the interface-rich sample. (a) XRD, (b) TEM and (c) the corresponding energy dispersive spectra (c) of the interface-rich sample.

As seen in Supplementary Fig. 24a, the interface-rich sample shows dispersive shape peaks due to the fast nucleation reaction in water without ammonia. The peak position of the interface-rich sample is consistent with the standard Cu (PDF # 85-1326) and Ag (PDF # 87-0717), proving that it remains a thermodynamically favored phase separation structure. TEM and the corresponding energy dispersive spectra (Supplementary Figs. 24b,c) prove the rich interfaces between Cu and Ag in the interface-rich sample and less exposed Ag sites in the composite.

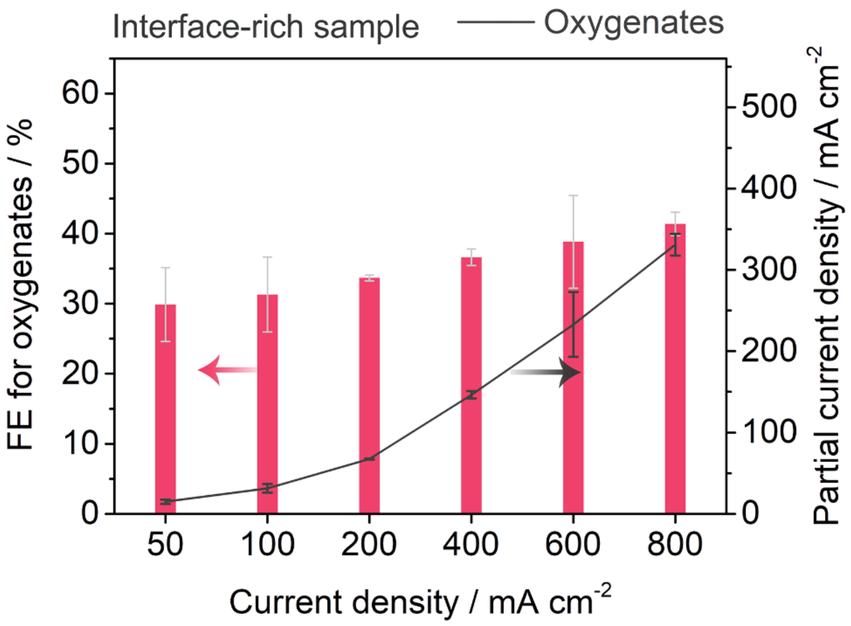


Supplementary Figure 25 | The CO-EC performance of the interface-rich sample. Current density-dependent Faradaic efficiency and partial current densities for oxygenates of the interface-rich sample for CO-EC. Error bars correspond to the Standard Deviation (SD) of three independent measurements.

The maximum FE for oxygenates of the interface-rich sample is ~40 %, which is weaker than that of Cu/30Ag, suggesting 1. That only abundant interfaces cannot remarkably improve the performance; 2. The Ag surface should be exposed in the composite, which is an important factor for performance enhancement.

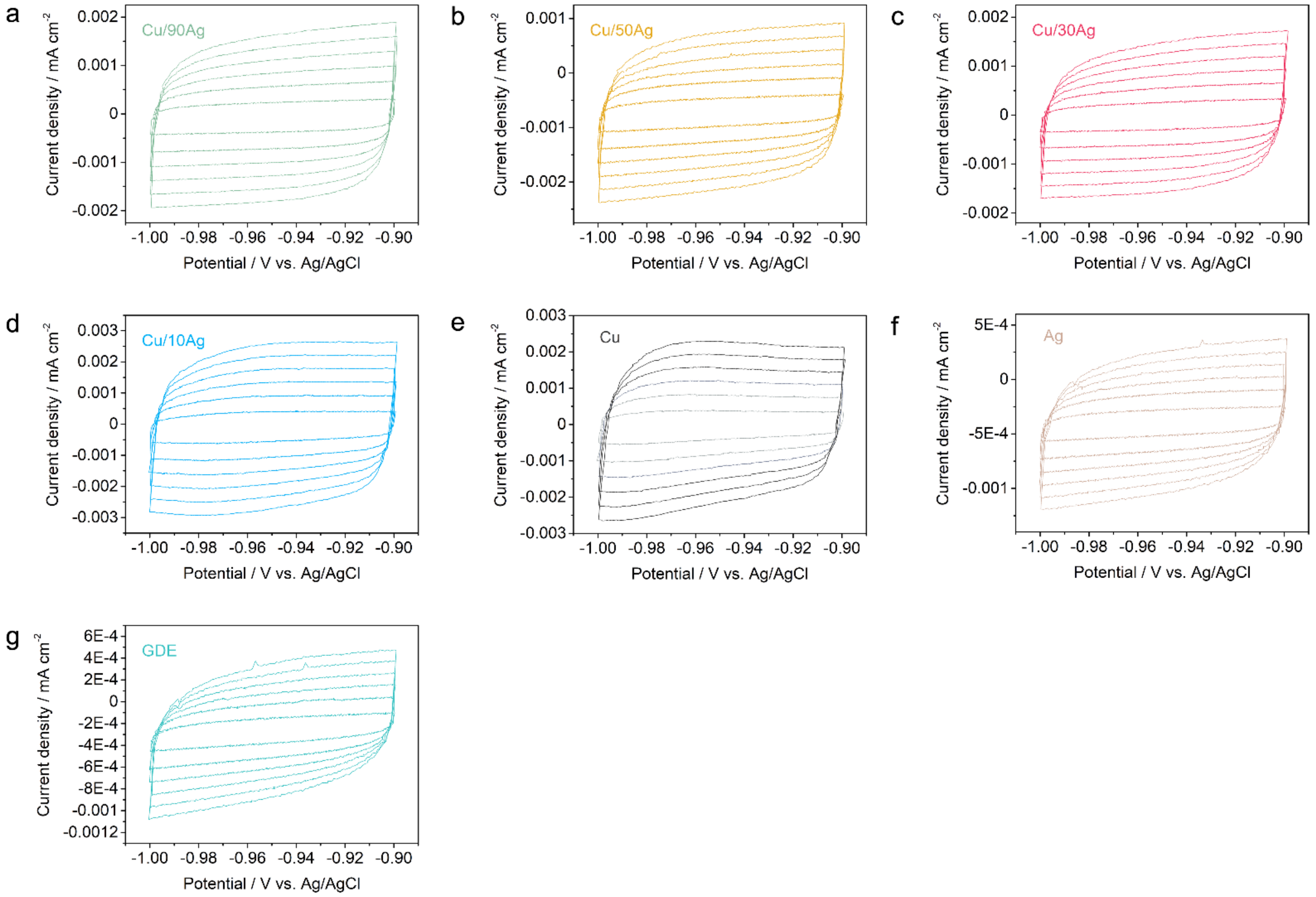


Supplementary Figure 26 | The cyclic voltammetry measurement. Cyclic voltammetry curves under different scan rates for Cu-Ag composites (a-d), Cu (e), Ag (f) and GDE (g).

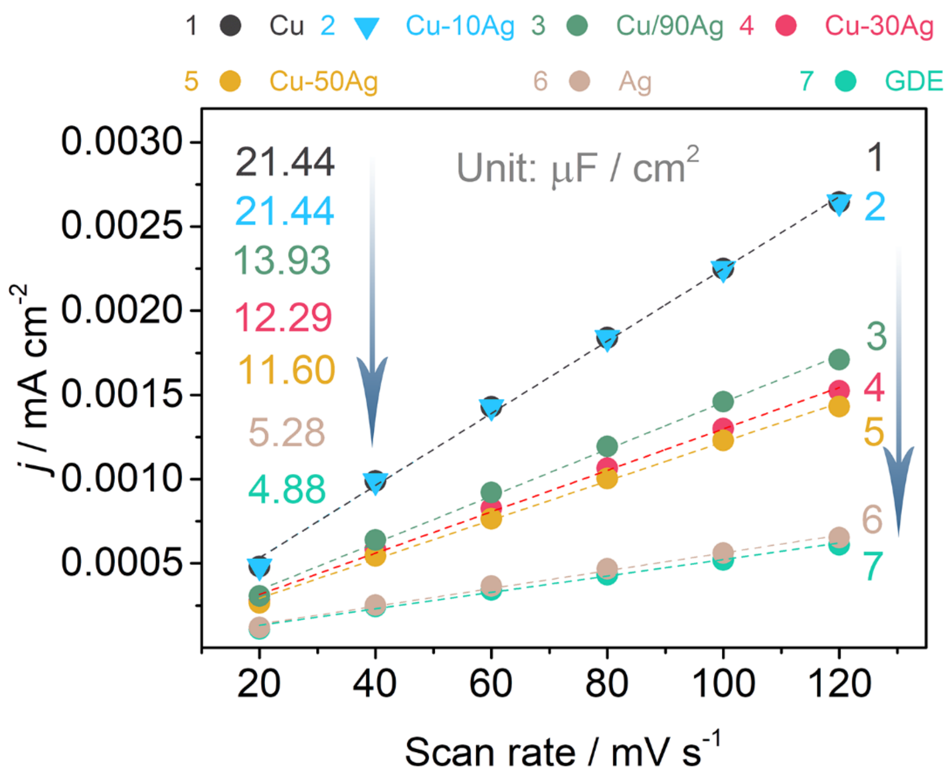


Supplementary Figure 27 | Double-layer capacitance measurements of Cu, Ag, Cu-Ag composites and GDE substrate.

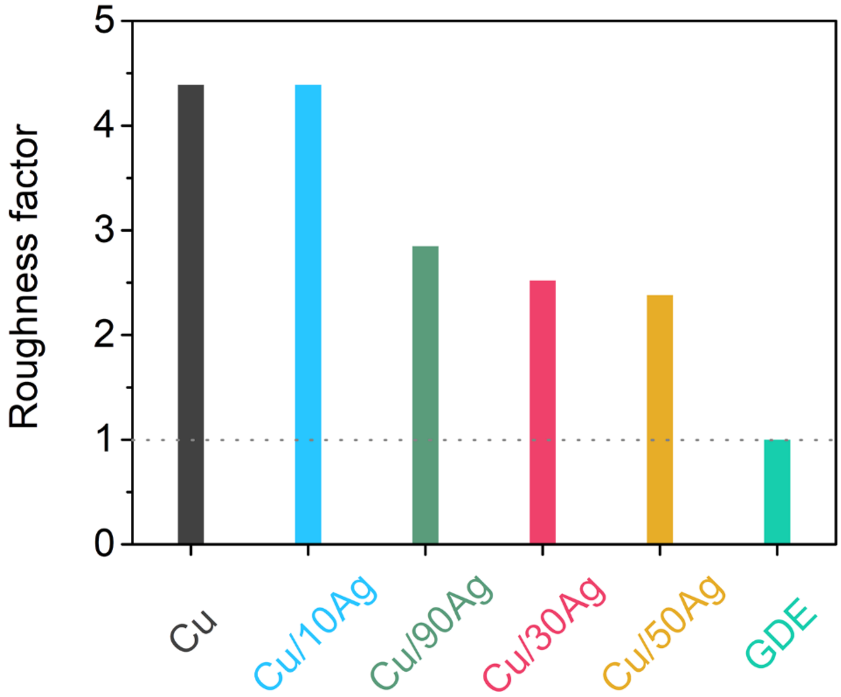


Supplementary Figure 28 | Roughness factors (RF) of the different samples. RF is defined by the double-layer capacitance of the sample over that of the GDE.

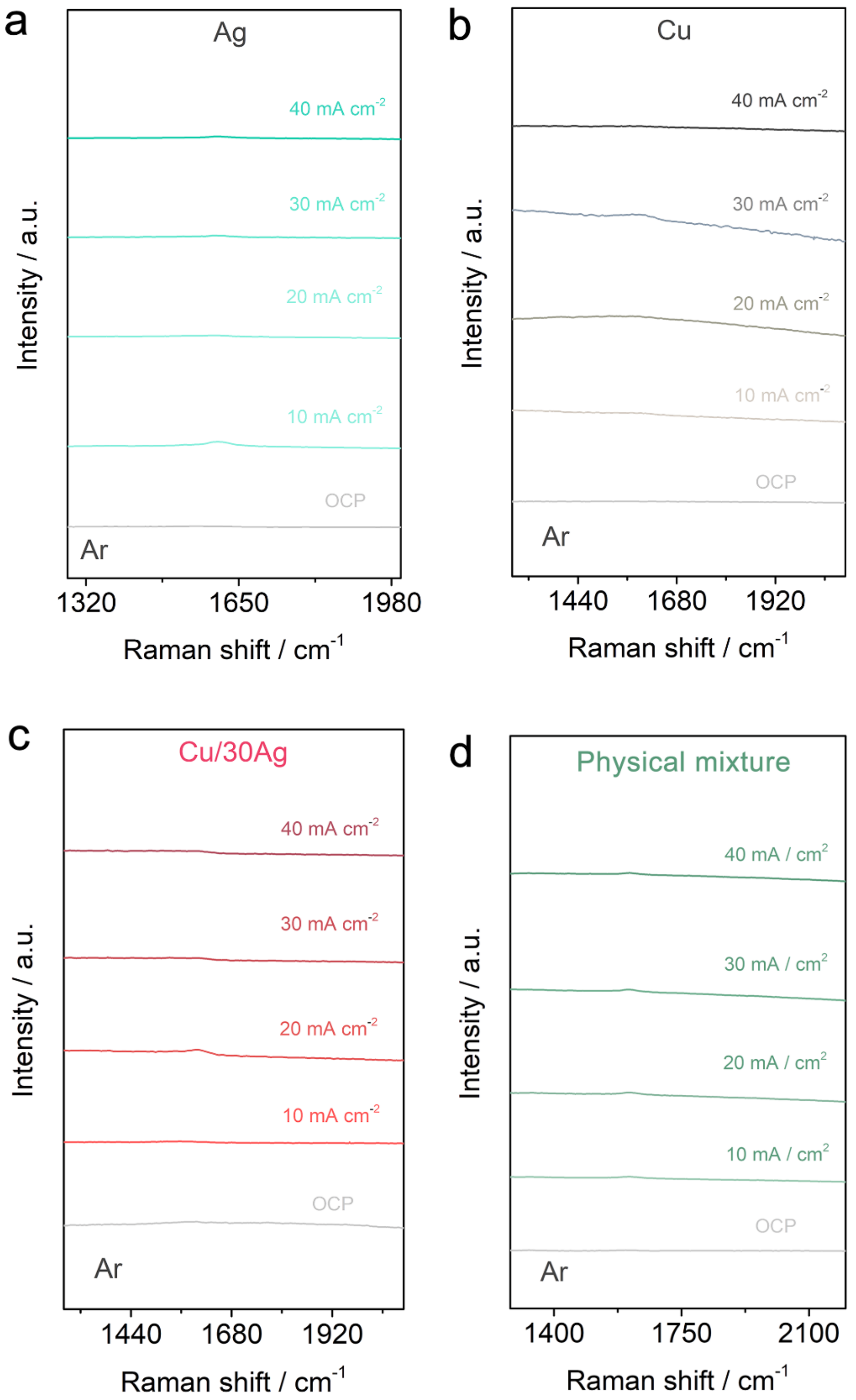


Supplementary Figure 29 | The *in situ* Raman spectra in Ar for comparison. *In situ* Raman spectra of Ag, Cu, Cu/30Ag and a physical mixture in an Ar atmosphere.

No Raman peak is found in the *in situ* Raman spectra of Ag, Cu, Cu/30Ag and the physical mixture in an Ar atmosphere.

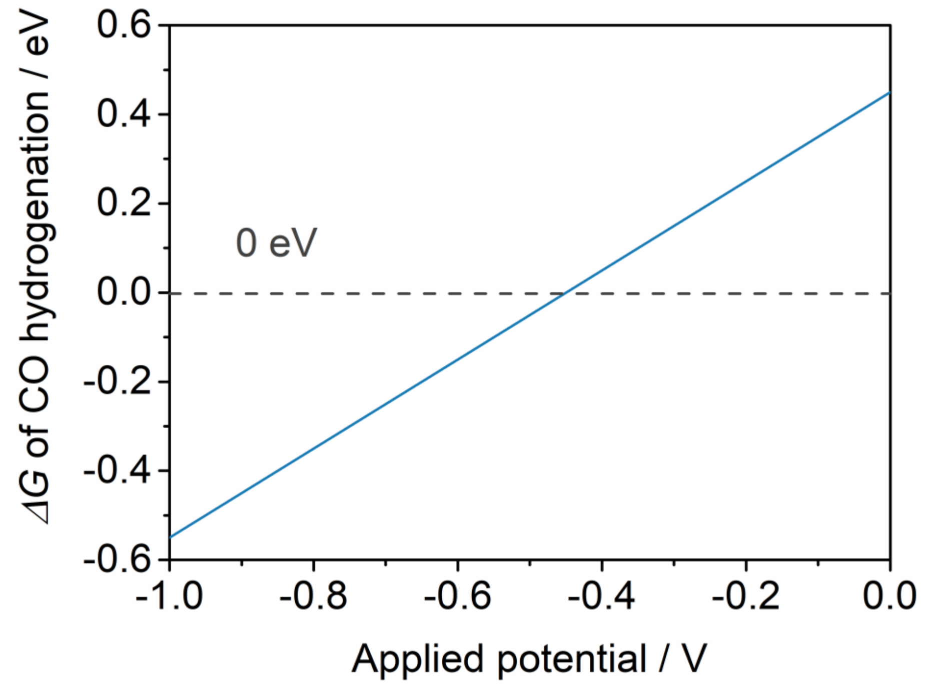


Supplementary Figure 30 │The Gibbs free energy change of CO hydrogenation with the change of the applied potentials on Ag(100).

Initially, the CO hydrogenation pathway leads to the formation of *HCO instead of *COH. As detailed in Supplementary Table 14, the disparity in adsorption Gibbs energy between *HCO and *COH ranges from -0.13 eV to -0.86 eV. Notably, the adsorption energy differences between Ag(100) and Cu(100), as well as Ag(111) and Cu(111), are -1.02 eV and -0.52 eV, respectively. These differences serve as the thermodynamic driving forces facilitating the transfer of *HCO from Ag to Cu. As indicated in the subsequent Supplementary Table 14, the CO hydrogenation steps on Ag and Cu surfaces exhibit energy ranges from 0.45 eV to 0.69 eV. Notably, the generation of *HCO can be influenced by applied potentials, altering the Gibbs free energy by -eU, as outlined in the Computational Hydrogen Electrode (CHE) model proposed by Nørskov[1]. The corresponding change in $\Delta G$ is illustrated in Supplementary Fig. 30, taking Ag(100) as an example. This analysis underscores that *CO hydrogenation step can thermodynamically transpire at negative potential below -0.45 V on Ag(100).

However, the CO dimerization reactions are thermodynamically implausible across all surfaces, as indicated by the Gibbs free energy changes ranging from 0.86 eV to 1.50 eV. According to Calle-Vallejo and Koper's work[2], CO dimerization step is promoted by the negatively charged CO dimer, ie., $C_2O_2^-$, the Gibbs free energy of which is further modulated by -eU. According to this framework, the thermodynamic feasibility of the CO dimerization step requires a minimum of -0.86 V. As a result, on Ag or Cu surfaces, the prevailing tendency for CO is to undergo hydrogenation to form *HCO rather than dimerization.

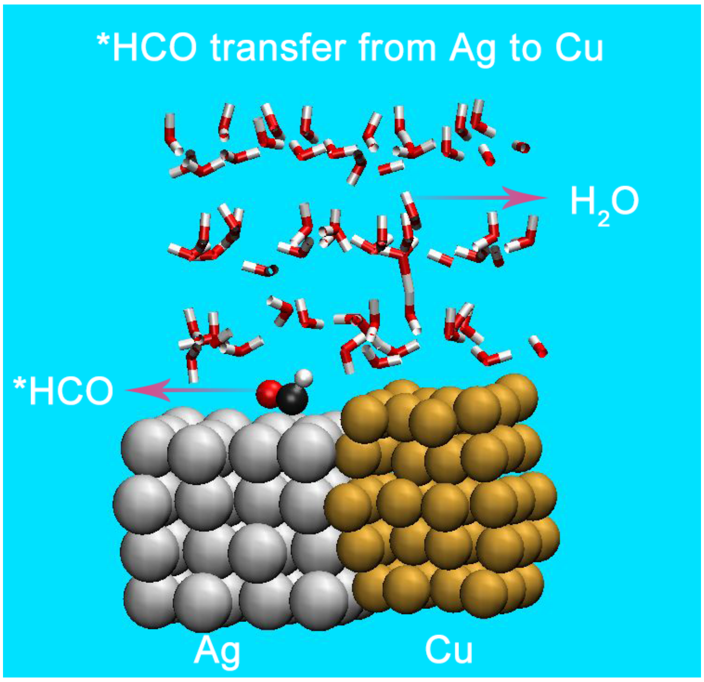


Supplementary Figure 31 | The legend for the video.

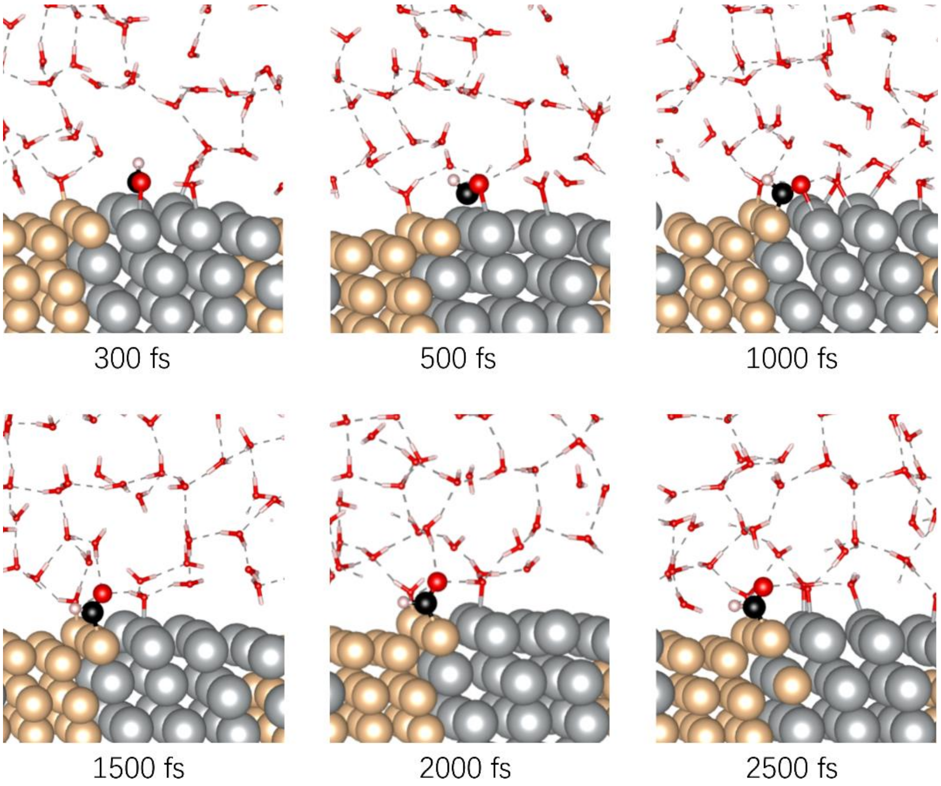


Supplementary Figure 32 │The typical snapshots of *HCO transfer of crossing the boundary of Ag(111) and Cu(111) with the boundary density of 1/9.

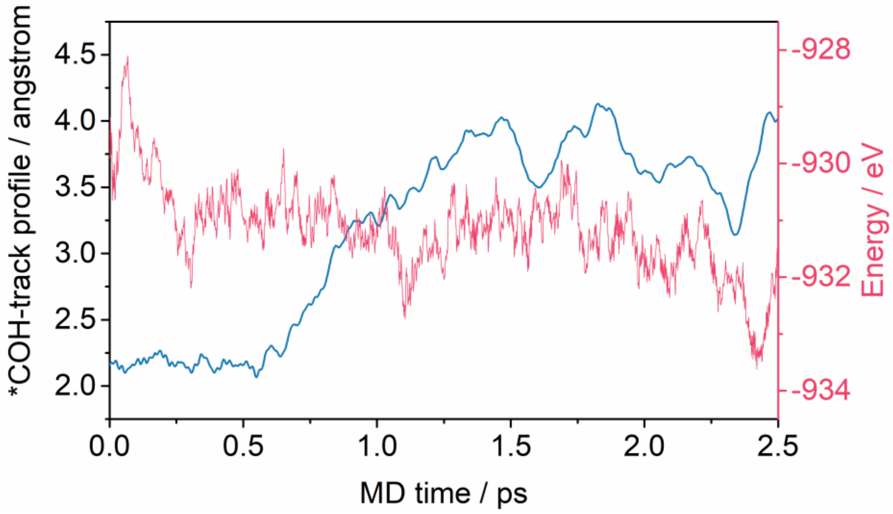


Supplementary Figure 33 | The trajectory profile of *HCO from Ag(111) to Cu(111) and the corresponding energy change.

As illustrated by the snapshots (Supplementary Fig. 32), the track profile and the corresponding energy change (Supplementary Fig. 33), the *HCO species also exhibits a spontaneous transfer phenomenon from Ag atoms to Cu atoms, which shows an exothermic adsorption energy of -0.52 eV for *HCO transfer from Ag(111) to Cu(111).

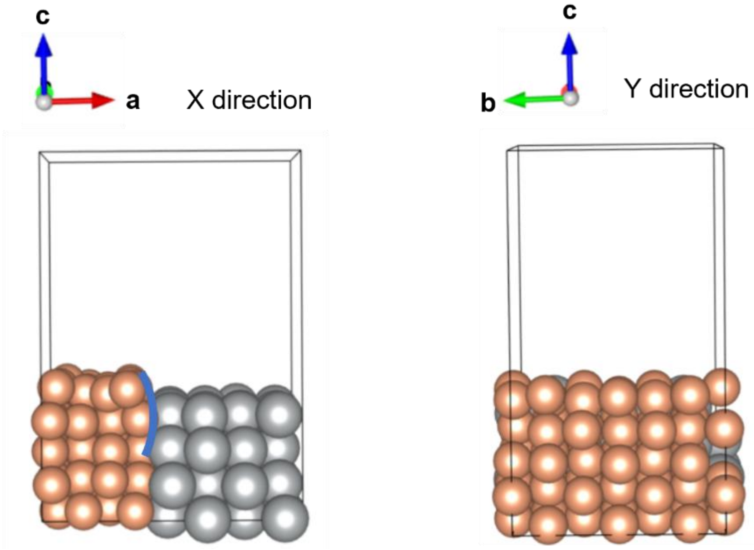


Supplementary Figure 34 | The interface structure of Cu(100) | Ag(100).

Given that the lattice constant of Ag surpasses that of Cu, there is anticipated extension or stress near the boundary region. To mitigate this, the Cu(100)|Ag(100) interface was constructed based on the fully relaxed bulk Cu(100)|Ag(100) with an identical atomic arrangement, aiming to minimize extension or stress, particularly in the boundary area. The calculated lattice constants for pure Ag and Cu are 4.126 Å and 3.615 Å, respectively. Consequently, the Cu(100)|Ag(100) interface model adopts modified lattice constants of 3.910 Å and 4.086 Å along the x and y directions, respectively. This corresponds to the stretching strain of 8.16 % and 13.02 % along x and y direction, respectively, compared to pure Cu, as well as the compressing strain of 5.23 % and 0.97 % along x and y, direction, respectively, compared to pure Ag. However, on the top layer of the interface model, the strain effect is minimized due to an ample space for Ag and Cu atoms to adjust to their most suitable lattice, resulting in the characteristic blue bending curve of atoms at the interface, as illustrated in the Supplementary Fig. 34.

**Supplementary Table 1.** The CO-EC performance of Cu at different applied current densities (unit: % for FE, mA $cm^{-2}$ for $j$).

| Products | 50 mA $cm^{-2}$<br>FE and $j$ | 100 mA $cm^{-2}$<br>FE and $j$ | 200 mA $cm^{-2}$<br>FE and $j$ | 400 mA $cm^{-2}$<br>FE and $j$ | 600 mA $cm^{-2}$<br>FE and $j$ | 800 mA $cm^{-2}$<br>FE and $j$ |
|---|---|---|---|---|---|---|
| **Hydrogen** | 50.41 ± 3.83<br>25.21 ± 1.92 | 32.81 ± 2.78<br>32.81 ± 2.78 | 27.21 ± 1.06<br>54.42 ± 2.12 | 24.46 ± 6.11<br>97.84 ± 24.44 | 34.09 ± 3.90<br>204.54 ± 23.40 | 17.81 ± 1.61<br>142.48 ± 12.88 |
| **Ethylene** | 21.12 ± 0.93<br>10.56 ± 0.47 | 30.45 ± 1.70<br>30.45 ± 1.70 | 35.82 ± 1.53<br>71.64 ± 3.06 | 36.29 ± 5.03<br>145.16 ± 20.12 | 31.85 ± 3.91<br>191.10 ± 23.46 | 42.38 ± 4.02<br>339.04 ± 32.16 |
| **Methane** | 0<br>0 | 0<br>0 | 0<br>0 | 0.13 ± 0.23<br>0.52 ± 0.92 | 0.42 ± 0.50<br>2.52 ± 3.00 | 0.07 ± 0.12<br>0.56 ± 0.96 |
| **Acetate anion** | 3.82 ± 2.67<br>1.91 ± 1.34 | 3.52 ± 0.13<br>3.52 ± 0.13 | 4.51 ± 0.55<br>9.02 ± 1.10 | 8.35 ± 4.36<br>33.40 ± 17.44 | 7.25 ± 1.13<br>43.50 ± 6.78 | 9.40 ± 0.51<br>75.20 ± 4.08 |
| **Acetaldehyde** | 0.75 ± 0.18<br>0.38 ± 0.09 | 0.90 ± 0.18<br>0.90 ±0.18 | 0.99 ± 0.29<br>1.98 ± 0.58 | 0.85 ± 0.32<br>3.40 ± 1.28 | 0.73 ± 0.14<br>4.38 ± 0.84 | 1.04 ± 0.44<br>8.32 ± 3.52 |
| **Propionaldehyde** | 1.62 ± 0.25<br>0.81 ± 0.13 | 1.46 ± 0.21<br>1.46 ± 0.21 | 0.93 ± 0.16<br>1.86 ± 0.32 | 0.35 ± 0.09<br>1.40 ± 0.36 | 0.20 ± 0.03<br>1.20 ± 0.18 | 0.31 ± 0.07<br>2.48 ± 0.56 |
| **Acetone** | 0.20 ± 0.09<br>0.10 ± 0.05 | 0.12 ± 0.01<br>0.12 ± 0.01 | 0.11 ± 0.01<br>0.22 ± 0.02 | 0.14 ± 0.03<br>0.56 ± 0.12 | 0.15 ± 0.03<br>0.90 ± 0.18 | 0.53 ± 0.19<br>4.24 ± 1.52 |
| **Ethanol** | 8.43 ± 0.89<br>4.22 ± 0.45 | 12.63 ± 0.28<br>12.63 ± 0.28 | 15.31 ± 1.21<br>30.62 ± 2.42 | 16.09 ± 1.90<br>64.36 ± 7.60 | 15.88 ± 0.54<br>95.28 ± 3.24 | 16.75 ± 1.35<br>134.00 ± 10.80 |
| **n-propanol** | 12.17 ± 0.88<br>6.09 ± 0.44 | 15.66 ± 1.42<br>15.66 ± 1.42 | 13.29 ± 0.56<br>26.58 ± 1.12 | 10.56 ± 3.69<br>42.24 ± 14.76 | 7.95 ± 1.66<br>47.70 ± 9.96 | 7.68 ± 1.35<br>61.44 ± 10.80 |
| **Ally Alcohol** | 1.85 ± 0.59<br>0.93 ± 0.30 | 2.76 ± 0.38<br>2.76 ± 0.38 | 2.69 ± 0.36<br>5.38 ± 0.72 | 2.08 ± 0.31<br>8.32 ± 1.24 | 1.49 ± 0.19<br>8.94 ± 1.14 | 1.54 ± 0.28<br>12.32 ± 2.24 |
| **1-butanol** | 0.08 ± 0.03<br>0.04 ± 0.02 | 0.04 ± 0.01<br>0.04 ± 0.01 | 0.03 ± 0.01<br>0.06 ± 0.02 | 0.01 ± 0.01<br>0.04 ± 0.04 | 0.01 ± 0.01<br>0.06 ± 0.06 | 0<br>0 |
| **Methanol** | 0.53 ± 0.22<br>0.27 ± 0.11 | 0.44 ± 0.16<br>0.44 ± 0.16 | 0.68 ± 0.43<br>1.36 ± 0.86 | 0.57 ± 0.56<br>2.28 ± 2.24 | 0.48 ± 0.32<br>2.88 ± 1.92 | 0.37 ± 0.13<br>2.96 ± 1.04 |
| **Oxygenates** | 29.46 ± 3.11<br>14.73 ± 1.56 | 37.52 ± 0.84<br>37.52 ± 0.84 | 38.55 ± 1.86<br>77.10 ± 3.72 | 39.00 ± 1.95<br>156.00 ± 7.80 | 34.14 ± 1.68<br>204.84 ± 10.08 | 37.62 ± 1.19<br>300.96 ± 9.52 |

**Supplementary Table 2.** The CO-EC performance of Cu/10Ag at different applied current densities (unit: % for FE, mA cm$^{-2}$ for $j$).

| Products | 50 mA cm$^{-2}$ FE and $j$ | 100 mA cm$^{-2}$ FE and $j$ | 200 mA cm$^{-2}$ FE and $j$ | 400 mA cm$^{-2}$ FE and $j$ | 600 mA cm$^{-2}$ FE and $j$ | 800 mA cm$^{-2}$ FE and $j$ |
|---|---|---|---|---|---|---|
| **Hydrogen** | 56.45 ± 3.74<br>28.23 ± 1.87 | 36.32 ± 3.90<br>36.32 ± 3.90 | 28.79 ± 6.56<br>57.58 ± 13.12 | 15.86 ± 0.70<br>63.44 ± 2.80 | 16.41 ± 2.11<br>98.46 ± 12.66 | 19.58 ± 1.02<br>156.64 ± 8.16 |
| **Ethylene** | 21.86 ± 2.04<br>10.93 ± 1.02 | 28.94 ± 2.60<br>28.94 ± 2.60 | 29.54 ± 3.56<br>59.08 ± 7.12 | 38.81 ± 0.22<br>155.24 ± 0.88 | 25.52 ± 2.38<br>153.12 ± 14.28 | 34.12 ± 1.55<br>272.96 ± 12.40 |
| **Methane** | 0<br>0 | 0<br>0 | 0<br>0 | 0.21 ± 0.05<br>0.84 ± 0.20 | 0.87 ± 0.77<br>5.22 ± 4.62 | 1.07 ± 0.53<br>8.56 ± 4.24 |
| **Acetate anion** | 2.22 ± 0.15<br>1.11 ± 0.08 | 5.95 ± 0.53<br>5.95 ± 0.53 | 13.37 ± 3.20<br>26.74 ± 6.40 | 12.17 ± 4.17<br>48.68 ± 16.68 | 31.54 ± 3.31<br>189.24 ± 19.86 | 24.61 ± 0.40<br>196.88 ± 3.20 |
| **Acetaldehyde** | 1.30 ± 0.86<br>0.65 ± 0.43 | 1.67 ± 0.45<br>1.67 ± 0.45 | 1.23 ± 0.60<br>2.46 ± 1.20 | 1.56 ± 0.11<br>6.24 ± 0.44 | 1.13 ± 0.08<br>6.78 ± 0.48 | 1.32 ± 0.07<br>10.56 ± 0.56 |
| **Propionaldehyde** | 0.11 ± 0.10<br>0.06 ± 0.05 | 0.78 ± 0.66<br>0.78 ± 0.66 | 0.50 ± 0.32<br>1.00 ± 0.64 | 0.38 ± 0.13<br>1.52 ± 0.52 | 0.18 ± 0.04<br>1.08 ± 0.24 | 0.25 ± 0.08<br>2.00 ± 0.64 |
| **Acetone** | 0.76 ± 0.03<br>0.38 ± 0.02 | 0.26 ± 0.25<br>0.26 ± 0.25 | 0.15 ± 0.02<br>0.30 ± 0.04 | 0.30 ± 0.05<br>1.20 ± 0.20 | 0.24 ± 0.02<br>1.44 ± 0.12 | 0.50 ± 0.08<br>4.00 ± 0.64 |
| **Ethanol** | 5.94 ± 0.74<br>2.97 ± 0.37 | 13.96 ± 2.51<br>13.96 ± 2.51 | 14.91 ± 3.34<br>29.82 ± 6.68 | 17.97 ± 0.90<br>71.88 ± 3.60 | 14.34 ± 1.21<br>86.04 ± 7.26 | 13.74 ± 1.93<br>109.92 ± 15.44 |
| **n-propanol** | 9.56 ± 0.69<br>4.78 ± 0.35 | 8.27 ± 2.53<br>8.27 ± 2.53 | 9.37 ± 2.37<br>18.74 ± 4.74 | 7.83 ± 2.58<br>31.32 ± 10.32 | 4.39 ± 1.08<br>26.34 ± 6.48 | 3.79 ± 1.48<br>30.32 ± 11.84 |
| **Ally Alcohol** | 1.75 ± 0.07<br>0.88 ± 0.04 | 2.34 ± 0.34<br>2.34 ± 0.34 | 2.46 ± 0.13<br>4.92 ± 0.26 | 2.09 ± 0.47<br>8.36 ± 1.88 | 1.63 ± 0.22<br>9.78 ± 1.32 | 1.15 ± 0.40<br>9.20 ± 3.20 |
| **1-butanol** | 0<br>0 | 0.01 ± 0.02<br>0.01 ± 0.02 | 0.01 ± 0.01<br>0.02 ± 0.02 | 0<br>0 | 0.01 ± 0<br>0.06 ± 0 | 0.00 ± 0.01<br>0.00 ± 0.08 |
| **Methanol** | 0.18 ± 0.03<br>0.09 ± 0.02 | 0.73 ± 0.42<br>0.73 ± 0.42 | 0.48 ± 0.45<br>0.96 ± 0.90 | 0.62 ± 0.36<br>2.48 ± 1.44 | 0.40 ± 0.10<br>2.40 ± 0.60 | 0.54 ± 0.05<br>4.32 ± 0.40 |
| **Oxygenates** | 21.83 ± 1.19<br>10.92 ± 0.60 | 33.96 ± 2.72<br>33.96 ± 2.72 | 42.48 ± 3.62<br>84.96 ± 7.24 | 42.92 ± 2.67<br>171.68 ± 10.68 | 53.86 ± 1.52<br>323.16 ± 9.12 | 45.92 ± 1.68<br>367.36 ± 13.44 |

**Supplementary Table 3.** The CO-EC performance of Cu/30Ag at different applied current densities (unit: % for FE, mA $cm^{-2}$ for *j*).

| Products | 50 mA $cm^{-2}$ FE and *j* | 100 mA $cm^{-2}$ FE and *j* | 200 mA $cm^{-2}$ FE and *j* | 400 mA $cm^{-2}$ FE and *j* | 600 mA $cm^{-2}$ FE and *j* | 800 mA $cm^{-2}$ FE and *j* |
|---|---|---|---|---|---|---|
| **Hydrogen** | 54.01 ± 1.69<br>27.01 ± 0.85 | 40.70 ± 3.00<br>40.70 ± 3.00 | 26.34 ± 2.41<br>52.68 ± 4.82 | 21.86 ± 5.41<br>87.44 ± 21.64 | 14.63 ± 1.55<br>87.78 ± 9.30 | 14.38 ± 0.53<br>115.04 ± 4.24 |
| **Ethylene** | 18.87 ± 0.68<br>9.44 ± 0.34 | 25.17 ± 5.39<br>25.17 ± 5.39 | 26.46 ± 1.78<br>52.92 ± 3.56 | 27.99 ± 2.78<br>111.96 ± 11.12 | 27.18 ± 1.04<br>163.08 ± 6.24 | 25.96 ± 0.85<br>207.68 ± 6.80 |
| **Methane** | 0<br>0 | 0<br>0 | 3.75 ± 4.86<br>7.50 ± 9.72 | 2.21 ± 2.09<br>8.84 ± 8.36 | 0.44 ± 0.13<br>2.64 ± 0.78 | 1.14 ± 1.20<br>9.12 ± 9.60 |
| **Acetate anion** | 7.43 ± 1.92<br>3.72 ± 0.96 | 14.78 ± 3.00<br>14.78 ± 3.00 | 17.32 ± 0.97<br>34.64 ± 1.94 | 23.06 ± 3.48<br>92.24 ± 13.92 | 34.84 ± 1.52<br>209.04 ± 9.12 | 30.21 ± 2.67<br>241.68 ± 21.36 |
| **Acetaldehyde** | 1.80 ± 0.33<br>0.90 ± 0.17 | 2.03 ± 0.12<br>2.03 ± 0.12 | 1.88 ± 0.61<br>3.76 ± 1.22 | 1.41 ± 0.16<br>5.64 ± 0.64 | 1.44 ± 0.16<br>8.64 ± 0.96 | 1.60 ± 0.40<br>12.80 ± 3.20 |
| **Propionaldehyde** | 1.05 ± 0.08<br>0.53 ± 0.04 | 0.05 ± 0.01<br>0.05 ± 0.01 | 0.15 ± 0.14<br>0.30 ± 0.28 | 0.12 ± 0.11<br>0.48 ± 0.44 | 0.06 ± 0.00<br>0.36 ± 0.00 | 0.38 ± 0.19<br>3.04 ± 1.52 |
| **Acetone** | 0.20 ± 0.13<br>0.10±0.07 | 0.91 ± 0.42<br>0.91 ± 0.42 | 0.36 ± 0.12<br>0.72 ± 0.24 | 0.36 ± 0.13<br>1.44 ± 0.52 | 0.28 ± 0.04<br>1.68 ± 0.24 | 0.45 ± 0.09<br>3.60 ± 0.72 |
| **Ethanol** | 10.72 ± 1.89<br>5.36 ± 0.95 | 11.44 ± 1.25<br>11.44 ± 1.25 | 14.44 ± 0.43<br>28.88 ± 0.86 | 16.19 ± 2.81<br>64.76 ± 11.24 | 15.49 ± 0.41<br>92.94 ± 2.46 | 19.33 ± 2.01<br>154.64 ± 16.08 |
| **n-propanol** | 4.57 ± 0.95<br>2.29 ± 0.48 | 3.99 ± 0.88<br>3.99 ± 0.88 | 5.14 ± 2.09<br>10.28 ± 4.18 | 4.01 ± 1.05<br>16.04 ± 4.20 | 4.85 ± 0.76<br>29.10 ± 4.56 | 6.60 ± 1.40<br>52.80 ± 11.2 |
| **Ally Alcohol** | 2.15 ± 0.59<br>1.08 ± 0.30 | 1.71 ± 0.15<br>1.71 ± 0.15 | 2.08 ± 0.59<br>4.16 ± 1.18 | 1.48 ± 0.38<br>5.92 ± 1.52 | 1.76 ± 0.26<br>10.56 ± 1.56 | 1.71 ± 0.15<br>13.68 ± 1.20 |
| **1-butanol** | 0.20 ± 0.28<br>0.10 ± 0.14 | 0<br>0 | 0.01 ± 0.02<br>0.02 ± 0.04 | 0<br>0 | 0<br>0 | 0<br>0 |
| **Methanol** | 0.72 ± 0.30<br>0.36 ± 0.15 | 0.44 ± 0.14<br>0.44 ± 0.14 | 0.44 ± 0.06<br>0.88 ± 0.12 | 0.81 ± 0.36<br>3.24 ± 1.44 | 0.43 ± 0.04<br>2.58 ± 0.24 | 0.50 ± 0.09<br>4.00 ± 0.72 |
| **Oxygenates** | 28.84 ± 1.06<br>14.42 ± 0.53 | 35.33 ± 1.97<br>35.33 ± 1.97 | 41.82 ± 1.46<br>83.64 ± 2.92 | 47.44 ± 1.91<br>189.76 ± 7.64 | 59.15 ± 2.05<br>354.90 ± 12.30 | 60.77 ± 1.52<br>486.16 ± 12.16 |

**Supplementary Table 4.** The CO-EC performance of Cu/50Ag at different applied current densities (unit: % for FE, mA cm$^{-2}$ for *j*).

| Products | 50 mA cm$^{-2}$<br>FE and *j* | 100 mA cm$^{-2}$<br>FE and *j* | 200 mA cm$^{-2}$<br>FE and *j* | 400 mA cm$^{-2}$<br>FE and *j* | 600 mA cm$^{-2}$<br>FE and *j* |
|---|---|---|---|---|---|
| **Hydrogen** | 48.49 ± 2.87<br>24.25 ± 1.44 | 36.22 ± 2.95<br>36.22 ± 2.95 | 22.31 ± 5.91<br>44.62 ± 11.82 | 15.21 ± 0.35<br>60.84 ± 1.40 | 23.72 ± 5.84<br>142.32 ± 35.04 |
| **Ethylene** | 20.77 ± 3.11<br>10.39 ± 1.56 | 26.44 ± 4.32<br>26.44 ± 4.32 | 25.74 ± 0.55<br>51.48 ± 1.1 | 25.47 ± 1.80<br>101.88 ± 7.20 | 25.25 ± 2.15<br>151.50 ± 12.90 |
| **Methane** | 0<br>0 | 0<br>0 | 5.13 ± 3.48<br>10.26 ± 6.96 | 4.89 ± 1.56<br>19.56 ± 6.24 | 16.44 ± 2.91<br>98.64 ± 17.46 |
| **Acetate anion** | 5.67 ± 1.70<br>2.84 ± 0.85 | 10.99 ± 2.06<br>10.99 ± 2.06 | 24.92 ± 1.95<br>49.84 ± 3.90 | 34.60 ± 0.11<br>138.40 ± 0.44 | 15.82 ± 0.68<br>94.92 ± 4.08 |
| **Acetaldehyde** | 1.75 ± 0.39<br>0.88 ± 0.2 | 1.87 ± 0.86<br>1.87 ± 0.86 | 1.33 ± 0.32<br>2.66 ± 0.64 | 1.81 ± 0.23<br>7.24 ± 0.92 | 1.05 ± 0.10<br>6.30 ± 0.60 |
| **Propionaldehyde** | 1.34 ± 0.51<br>0.67 ± 0.26 | 0.33 ± 0.45<br>0.33 ± 0.45 | 0.11 ± 0.18<br>0.22 ± 0.36 | 0.02 ± 0.02<br>0.08 ± 0.08 | 0.01 ± 0.01<br>0.06 ± 0.06 |
| **Acetone** | 0.23 ± 0.06<br>0.12 ± 0.03 | 0.34 ± 0.23<br>0.34 ± 0.23 | 0.65 ± 0.25<br>1.30 ± 0.50 | 0.46 ± 0.02<br>1.84 ± 0.08 | 0.60 ± 0.14<br>3.60 ± 0.84 |
| **Ethanol** | 11.73 ± 1.67<br>5.87 ± 0.84 | 13.16 ± 3.27<br>13.16 ± 3.27 | 13.69 ± 1.86<br>27.38 ± 3.72 | 12.85 ± 0.61<br>51.40 ± 2.44 | 13.67 ± 0.27<br>82.02 ± 1.62 |
| **n-propanol** | 7.67 ± 4.31<br>3.84 ± 2.16 | 6.96 ± 2.00<br>6.96 ± 2.00 | 3.55 ± 0.82<br>7.10 ± 1.64 | 2.29 ± 0.66<br>9.16 ± 2.64 | 1.52 ± 0.08<br>9.12 ± 0.48 |
| **Ally Alcohol** | 2.18 ± 0.62<br>1.09 ± 0.31 | 2.50 ± 0.33<br>2.50 ± 0.33 | 1.73 ± 0.31<br>3.46 ± 0.62 | 0.94 ± 0.23<br>3.76 ± 0.92 | 0.65 ± 0.08<br>3.90 ± 0.48 |
| **1-butanol** | 0.28 ± 0.42<br>0.14 ± 0.21 | 0<br>0 | 0.01 ± 0.01<br>0.02 ± 0.02 | 0<br>0 | 0.01 ± 0.01<br>0.06 ± 0.06 |
| **Methanol** | 0.49 ± 0.34<br>0.25 ± 0.17 | 0.59 ± 0.56<br>0.59 ± 0.56 | 0.54 ± 0.19<br>1.08 ± 0.38 | 0.78 ± 0.08<br>3.12 ± 0.32 | 0.88 ± 0.07<br>5.28 ± 0.42 |
| **Oxygenates** | 31.34 ± 2.73<br>15.67 ± 1.37 | 36.74 ± 2.93<br>36.74 ± 2.93 | 46.52 ± 3.53<br>93.04 ± 7.06 | 53.76 ± 1.68<br>215.04 ± 6.72 | 34.21 ± 1.15<br>205.26 ± 6.90 |

**Supplementary Table 5.** The CO-EC performance of Cu/90Ag at different applied current densities (unit: % for FE, mA cm$^{-2}$ for *j*).

| Products | 50 mA cm$^{-2}$<br>FE and *j* | 100 mA cm$^{-2}$<br>FE and *j* | 200 mA cm$^{-2}$<br>FE and *j* |
|---|---|---|---|
| **Hydrogen** | 46.68 ± 5.63<br>23.34 ± 2.82 | 29.72 ± 2.95<br>29.72 ± 2.95 | 41.06 ± 5.03<br>82.12 ± 10.06 |
| **Ethylene** | 18.22 ± 2.66<br>9.11 ± 1.33 | 23.80 ± 1.86<br>23.80 ± 1.86 | 24.85 ± 4.40<br>49.70 ± 8.80 |
| **Methane** | 0.37 ± 0.64<br>0.19 ± 0.32 | 2.65 ± 1.31<br>2.65 ± 1.31 | 0.47 ± 0.81<br>0.94 ± 1.62 |
| **Acetate anion** | 8.80 ± 0.25<br>4.40 ± 0.13 | 13.03 ± 1.44<br>13.03 ± 1.44 | 12.27 ± 3.13<br>24.54 ± 6.26 |
| **Acetaldehyde** | 2.07 ± 0.55<br>1.04 ± 0.28 | 1.76 ± 0.67<br>1.76 ± 0.67 | 1.50 ± 0.40<br>3.00 ± 0.80 |
| **Propionaldehyde** | 0.21 ± 0.22<br>0.11 ± 0.11 | 0.48 ± 0.25<br>0.48 ± 0.25 | 0.30 ± 0.26<br>0.60 ± 0.52 |
| **Acetone** | 0.92 ± 0.37<br>0.46 ± 0.19 | 0.57 ± 0.20<br>0.57 ± 0.20 | 0.43 ± 0.36<br>0.86 ± 0.72 |
| **Ethanol** | 15.57 ± 1.48<br>7.79 ± 0.74 | 19.20 ± 3.25<br>19.20 ± 3.25 | 13.48 ± 2.55<br>26.96 ± 5.10 |
| **n-propanol** | 3.89 ± 0.34<br>1.95 ± 0.17 | 4.59 ± 0.87<br>4.59 ± 0.87 | 4.39 ± 2.97<br>8.78 ± 5.94 |
| **Ally Alcohol** | 2.27 ± 0.12<br>1.14 ± 0.06 | 2.20 ± 0.21<br>2.20 ± 0.21 | 1.74 ± 0.83<br>3.48 ± 1.66 |
| **1-butanol** | 0.03 ± 0.05<br>0.02 ± 0.03 | 0.03 ± 0.03<br>0.03 ± 0.03 | 0<br>0 |
| **Methanol** | 0.42 ± 0.06<br>0.21 ± 0.03 | 0.46 ± 0.04<br>0.46 ± 0.04 | 0.91 ± 0.61<br>1.82 ± 1.22 |
| **Oxygenates** | 34.17± 1.05<br>17.09 ± 0.53 | 42.33± 0.17<br>42.33 ± 0.17 | 35.01± 0.69<br>70.02 ± 1.38 |

**Supplementary Table 6.** The CO-EC performance of Ag for oxygenates generation at 800 mA $cm^{-2}$ (unit: % for FE, mA $cm^{-2}$ for $j$).

| Products | 800 mA $cm^{-2}$<br>FE and $j$ |
| --- | --- |
| **Acetate anion** | 1.17 ± 0.02<br>9.36 ± 0.16 |
| **Acetaldehyde** | 0.06 ± 0.01<br>0.48 ± 0.08 |
| **Propionaldehyde** | 0<br>0 |
| **Acetone** | 0.02 ± 0.00<br>0.16 ± 0.00 |
| **Ethanol** | 0.38 ± 0.02<br>3.04 ± 0.16 |
| **n-propanol** | 0.03 ± 0.00<br>0.24 ± 0.00 |
| **Ally Alcohol** | 0.01 ± 0.00<br>0.08 ± 0.00 |
| **1-butanol** | 0<br>0 |
| **Methanol** | 0<br>0 |
| **Oxygenates** | 1.66 ± 0.05<br>13.28 ± 0.40 |

**Supplementary Table 7.** The CO-EC performance of Cu/30Ag at 800 mA $cm^{-2}$ under a CO flow rate of 5 SCCM (unit: % for FE, mA $cm^{-2}$ for $j$).

| Products | 5 SCCM<br>FE and $j$ |
|---|---|
| **Hydrogen** | 26.12 ± 1.27<br>208.96 ± 10.16 |
| **Ethylene** | 13.29 ± 2.24<br>106.32 ± 17.92 |
| **Methane** | 5.19 ± 2.61<br>41.52 ± 20.88 |
| **Acetate anion** | 25.96 ± 1.75<br>207.68 ± 14.00 |
| **Acetaldehyde** | 0.75 ± 0.20<br>6 ± 1.6 |
| **Propionaldehyde** | 0.11 ± 0.01<br>0.88 ± 0.08 |
| **Acetone** | 0.29 ± 0.09<br>2.32 ± 0.72 |
| **Ethanol** | 26.04 ± 3.11<br>208.32 ± 24.88 |
| **n-propanol** | 2.15 ± 0.36<br>17.20 ± 2.88 |
| **Ally Alcohol** | 0.47 ± 0.12<br>3.76 ± 0.96 |
| **1-butanol** | 0<br>0 |
| **Methanol** | 0.29 ± 0.06<br>2.32 ± 0.48 |
| **Oxygenates** | 56.07 ± 2.35<br>448.56 ± 13.80 |

**Supplementary Table 8.** The CO-EC performance of Cu/30Ag at 1000 mA cm$^{-2}$ electrolysis with different flow rates of electrolyte (unit: % for FE, mA cm$^{-2}$ for *j*).

| Products | 10 SCCM<br>FE and *j* | 50 SCCM<br>FE and *j* | 100 SCCM<br>FE and *j* |
|---|---|---|---|
| **Hydrogen** | 29.30 ± 3.90 | 22.30 ± 4.41 | 10.95 ± 1.28 |
| | 293.00 ± 39.00 | 223.00 ± 44.10 | 109.50 ± 12.80 |
| **Ethylene** | 19.52 ± 3.00 | 21.74 ± 0.30 | 23.86 ± 1.02 |
| | 195.20 ± 30.00 | 217.40 ± 3.00 | 238.60 ± 10.20 |
| **Methane** | 14.89 ± 1.12 | 11.82 ± 1.77 | 4.64 ± 1.41 |
| | 148.90 ± 11.20 | 118.20 ± 17.70 | 46.40 ± 14.10 |
| **Acetate anion** | 17.61 ± 2.32 | 18.89 ± 4.55 | 32.81 ± 0.38 |
| | 176.10 ± 23.20 | 188.90 ± 45.50 | 328.10 ± 3.80 |
| **Acetaldehyde** | 1.16 ± 0.13 | 1.84 ± 0.30 | 3.31 ± 0.77 |
| | 11.60 ± 1.30 | 18.40 ± 3.00 | 33.10 ± 7.70 |
| **Propionaldehyde** | 0.19 ± 0.00 | 0.18 ± 0.06 | 0.30 ± 0.07 |
| | 1.90 ± 0.00 | 1.80 ± 0.60 | 3.00 ± 0.70 |
| **Acetone** | 0.38 ± 0.05 | 0.56 ± 0.04 | 0.69 ± 0.17 |
| | 3.80 ± 0.50 | 5.60 ± 0.40 | 6.90 ± 1.70 |
| **Ethanol** | 14.06 ± 0.39 | 19.69 ± 1.16 | 19.59 ± 2.19 |
| | 140.60 ± 3.90 | 196.90 ± 11.60 | 195.90 ± 21.90 |
| **n-propanol** | 1.57 ± 0.22 | 2.06 ± 0.25 | 2.27 ± 0.41 |
| | 15.70 ± 2.20 | 20.60 ± 2.50 | 22.70 ± 4.10 |
| **Ally Alcohol** | 0.49 ± 0.05 | 0.74 ± 0.11 | 0.93 ± 0.06 |
| | 4.90 ± 0.50 | 7.40 ± 1.10 | 9.30 ± 0.60 |
| **1-butanol** | 0 | 0 | 0 |
| | 0 | 0 | 0 |
| **Methanol** | 0.61 ± 0.06 | 0.96 ± 0.06 | 1.24 ± 0.30 |
| | 6.10 ± 0.060 | 9.60 ± 0.06 | 12.40 ± 0.30 |
| **Oxygenates** | 36.08± 2.64 | 44.92± 3.20 | 61.15± 3.02 |
| | 360.80 ± 26.40 | 449.20 ± 32.00 | 611.50 ± 30.20 |

**Supplementary Table 9.** The CO-EC performance of Cu/30Ag at 1200, and 1400 mA cm$^{-2}$ electrolysis with a flow rate of 100 mL min$^{-1}$ electrolyte (unit: % for FE, mA cm$^{-2}$ for $j$).

| Products | 1200 mA cm$^{-2}$<br>FE and $j$ | 1400 mA cm$^{-2}$<br>FE and $j$ |
|---|---|---|
| **Hydrogen** | 9.41 ± 0.3<br>112.92 ± 3.6 | 13.7 ± 1.12<br>191.8 ± 15.68 |
| **Ethylene** | 22.22 ± 0.73<br>266.64 ± 8.76 | 20.92 ± 0.93<br>292.88 ± 13.02 |
| **Methane** | 1.99 ± 1.12<br>23.88 ± 13.44 | 5.05 ± 0.42<br>70.7 ± 5.88 |
| **Acetate anion** | 34.43 ± 1.34<br>413.16 ± 16.08 | 27.28 ± 2.65<br>381.92 ± 37.1 |
| **Acetaldehyde** | 1.46 ± 0.07<br>17.52 ± 0.84 | 1.11 ± 0.04<br>15.54 ± 0.56 |
| **Propionaldehyde** | 0.01 ± 0.00<br>0.12 ± 0.00 | 0.01 ± 0.00<br>0.14 ± 0.00 |
| **Acetone** | 0.78 ± 0.08<br>9.36 ± 0.96 | 0.77 ± 0.06<br>10.78 ± 0.84 |
| **Ethanol** | 24.51 ± 0.37<br>294.12 ± 4.44 | 24.97 ± 0.67<br>349.58 ± 9.38 |
| **n-propanol** | 3.19 ± 0.51<br>38.28 ± 6.12 | 2.28 ± 0.12<br>31.92 ± 1.68 |
| **Ally Alcohol** | 1.08 ± 0.22<br>12.96 ± 2.64 | 0.66 ± 0.02<br>9.24 ± 0.28 |
| **1-butanol** | 0<br>0 | 0<br>0 |
| **Methanol** | 1.33 ± 0.16<br>15.96 ± 1.92 | 1.32 ± 0.12<br>18.48 ± 1.68 |
| **Oxygenates** | 66.81 ± 1.99<br>801.72 ± 23.88 | 58.41 ± 3.56<br>817.74 ± 49.84 |

**Supplementary Table 10.** The performance list of typical catalysts used for CO-to-oxygenate electroconversion at their respective optimized FE points and ambient conditions.

| Samples | Partial current density / mA $cm^{-2}$ | $FE_{Oxygenates}$ / % | Refs. |
|---|---|---|---|
| **Cu/30 %Ag** | **801.72** | **66.81** | **This work** |
| **OD-Cu** | 84 | 42 | Nat. Catal., 1, 748 (2018) |
| **Ag-Ru-Cu** | 300 | 60 | Nat. Energy, 7, 170 (2022) |
| **CuPd** | 480 | 72 | Nat. Catal., 5, 251 (2022) |
| **CuO** | 373 | 34 | Nat. Catal., 2, 1124 (2019) |
| **CuO nanosheet** | 550 | 55 | Nat. Nanotechnol., 18, 299 (2023) |
| **$Ag_{1\ \%}$-$Cu_2O$** | 141 | 71 | Nat. Synth., 2, 448 (2023) |
| **Cu nanosheets** | 137 | 50 | Nat. Catal., 2, 423 (2019) |
| **$Ag_2Cu_2O_3$** | 360 | 60 | Energy. Environ. Sci., 13, 2993 (2020) |
| **$CuPd_{0.007}$** | 362 | 52 | Nat. Commun., 11, 3685 (2020) |
| **$Cu(OD)_{0.8}Ag_{0.2}$** | 120 | 76.9 | Nat. Commun., 14, 698 (2023) |
| **OD-Cu/MgAlNS** | 200 | 40 | Angew. Chem. Int. Ed., 62, e202217252 (2023) |
| **Cu NC** | 180 | 40 | PNAS, 118, e2010868118 (2021) |
| **$Cu/Cu_2O$** | 119.4 | 74 | Nat. Commun., 14, 501 (2023) |
| **$Cu_{49}Pd_{51}$** | 222 | 73 | ACS Catal., 12, 5275 (2022) |

**Supplementary Table 11.** The performance list of Cu/30Ag used for CO-to-oxygenate electroconversion in MEA (unit: % for FE, mA cm$^{-2}$ for $j$).

| Time/h | **Acetate anion /FE and *j*** | **Acetaldehyde/FE and *j*** | **Propionaldehyde/FE and *j*** | **Acetone/FE and *j*** | **Ethanol/FE and *j*** | **n-propanol/FE and *j*** | **Ally Alcohol/FE and *j*** | **1-butanol/FE and *j*** | **Methanol/FE and *j*** | **Oxygenates/FE and *j*** |
|---|---|---|---|---|---|---|---|---|---|---|
| 1 | 21.76 | 1.08 | 0.33 | 0.71 | 19.80 | 4.82 | 0.72 | 0 | 0.44 | 49.66 |
| | 108.80 | 5.40 | 1.65 | 3.55 | 99.00 | 24.10 | 3.60 | 0 | 2.20 | 248.30 |
| 2 | 26.8.0 | 1.10 | 0.29 | 0.74 | 21.00 | 4.19 | 0.73 | 0 | 0.56 | 55.42 |
| | 134.00 | 5.50 | 1.45 | 3.70 | 105.00 | 20.95 | 3.65 | 0 | 2.80 | 277.10 |
| 3 | 27.83 | 0.97 | 0.25 | 0.78 | 19.17 | 3.33 | 0.63 | 0 | 0.55 | 53.51 |
| | 139.15 | 4.85 | 1.25 | 3.90 | 95.85 | 16.65 | 3.15 | 0 | 2.75 | 267.55 |
| 4 | 28.81 | 0.94 | 0.21 | 0.71 | 19.08 | 2.93 | 0.6 | 0 | 0.56 | 53.84 |
| | 144.05 | 4.70 | 1.05 | 3.55 | 95.40 | 14.65 | 3.00 | 0 | 2.80 | 269.20 |
| 5 | 32.49 | 0.99 | 0.21 | 0.80 | 19.71 | 2.78 | 0.61 | 0 | 0.67 | 58.26 |
| | 162.45 | 4.95 | 1.05 | 4.00 | 98.55 | 13.9 | 3.05 | 0 | 3.35 | 291.3 |
| 6 | 32.4 | 0.8 | 0.07 | 0.67 | 16.93 | 2.16 | 0.47 | 0 | 0.61 | 54.09 |
| | 162.00 | 4.00 | 0.35 | 3.35 | 84.65 | 10.80 | 2.35 | 0 | 3.05 | 270.45 |
| 7 | 33.81 | 0.88 | 0.17 | 0.82 | 17.35 | 2.07 | 0.48 | 0 | 0.67 | 56.26 |
| | 169.05 | 4.40 | 0.85 | 4.10 | 86.75 | 10.35 | 2.40 | 0 | 3.35 | 281.30 |
| 8 | 31.21 | 0.82 | 0.16 | 0.76 | 16.63 | 1.87 | 0.44 | 0 | 0.63 | 52.52 |
| | 156.05 | 4.10 | 0.80 | 3.80 | 83.15 | 9.35 | 2.20 | 0 | 3.15 | 262.60 |
| 9 | 30.81 | 0.83 | 0.15 | 0.8 | 16.26 | 1.72 | 0.42 | 0 | 0.65 | 51.64 |
| | 154.05 | 4.15 | 0.75 | 4.00 | 81.30 | 8.60 | 2.10 | 0 | 3.25 | 258.20 |
| 10 | 34.36 | 0.83 | 0.15 | 0.81 | 17.26 | 1.75 | 0.45 | 0 | 0.69 | 56.30 |
| | 171.80 | 4.15 | 0.75 | 4.05 | 86.30 | 8.75 | 2.25 | 0 | 3.45 | 281.50 |
| 11 | 33.07 | 0.76 | 0.13 | 0.71 | 16.2 | 1.55 | 0.4 | 0 | 0.64 | 53.44 |
| | 165.35 | 3.80 | 0.65 | 3.55 | 81.00 | 7.75 | 2.00 | 0 | 3.20 | 267.20 |
| 12 | 35.47 | 0.72 | 0.07 | 0.91 | 16.2 | 1.58 | 0.39 | 0 | 0.62 | 55.96 |
| | 177.35 | 3.60 | 0.35 | 4.55 | 81.00 | 7.90 | 1.95 | 0 | 3.10 | 279.80 |
| 13 | 35.56 | 0.72 | 0.06 | 0.73 | 16.48 | 1.57 | 0.39 | 0 | 0.62 | 56.13 |
| | 177.80 | 3.60 | 0.30 | 3.65 | 82.40 | 7.85 | 1.95 | 0 | 3.10 | 280.65 |
| 14 | 34.18 | 0.63 | 0.05 | 0.64 | 15.43 | 1.21 | 0.31 | 0 | 0.56 | 53.00 |
| | 170.9 | 3.15 | 0.25 | 3.20 | 77.15 | 6.05 | 1.55 | 0 | 2.80 | 265.00 |
| 15 | 34.89 | 0.63 | 0.05 | 0.78 | 15.2 | 1.18 | 0.31 | 0 | 0.56 | 53.58 |
| | 174.45 | 3.15 | 0.25 | 3.90 | 76.00 | 5.90 | 1.55 | 0 | 2.80 | 267.9 |

**Supplementary Table 11.** The performance list of Cu/30Ag used for CO-to-oxygenates electro-conversion in MEA (unit: % for FE, mA $cm^{-2}$ for *j*) (Continued).

| Time / h | **Acetate anion /FE and *j*** | **Acetaldehyde/FE and *j*** | **Propionaldehyde/FE and *j*** | **Acetone/FE and *j*** | **Ethanol/FE and *j*** | **n-propanol/FE and *j*** | **Ally Alcohol/FE and *j*** | **1-butanol/FE and *j*** | **Methanol/FE and *j*** | **Oxygenates/FE and *j*** |
|---|---|---|---|---|---|---|---|---|---|---|
| 16 | 37.12 | 0.72 | 0.05 | 0.79 | 16.37 | 1.27 | 0.35 | 0 | 0.68 | 57.34 |
| | 185.60 | 3.60 | 0.25 | 3.95 | 81.85 | 6.35 | 1.75 | 0 | 3.40 | 286.70 |
| 22 | 34.13 | 0.76 | 0.05 | 0.88 | 17.18 | 1.14 | 0.31 | 0 | 0.82 | 55.27 |
| | 170.65 | 3.80 | 0.25 | 4.40 | 85.90 | 5.70 | 1.55 | 0 | 4.10 | 276.35 |
| 24 | 33.84 | 0.58 | 0.04 | 0.81 | 13.95 | 0.90 | 0.26 | 0 | 0.65 | 51.03 |
| | 169.20 | 2.90 | 0.20 | 4.05 | 69.75 | 4.50 | 1.30 | 0 | 3.25 | 255.15 |
| 26 | 33.99 | 0.53 | 0.04 | 0.82 | 13.82 | 0.84 | 0.26 | 0 | 0.61 | 50.90 |
| | 169.95 | 2.65 | 0.20 | 4.10 | 69.10 | 4.20 | 1.30 | 0 | 3.05 | 254.50 |
| 28 | 32.90 | 0.53 | 0.05 | 0.92 | 13.70 | 1.41 | 0.27 | 0 | 0.65 | 50.44 |
| | 164.50 | 2.65 | 0.25 | 4.60 | 68.50 | 7.05 | 1.35 | 0 | 3.25 | 252.20 |

**Supplementary Table 12.** The CO-EC performance of the physical mixture sample at different applied current densities (unit: % for FE, mA $cm^{-2}$ for *j*).

| Products | 50 mA $cm^{-2}$ | 100 mA $cm^{-2}$ | 200 mA $cm^{-2}$ | 400 mA $cm^{-2}$ | 600 mA $cm^{-2}$ | 800 mA $cm^{-2}$ |
|---|---|---|---|---|---|---|
| | FE and *j* | FE and *j* | FE and *j* | FE and *j* | FE and *j* | FE and *j* |
| **Hydrogen** | 49.27 ± 1.46 | 48.96 ± 2.48 | 33.77 ± 4.18 | 23.23 ± 2.29 | 24.21 ± 4.17 | 24.75 ± 1.21 |
| | 24.64 ± 0.73 | 48.96 ± 2.48 | 67.54 ± 8.36 | 92.92 ± 9.16 | 145.26 ± 25.02 | 198.00 ± 9.68 |
| **Ethylene** | 18.1 ± 0.62 | 19.68 ± 1.13 | 26.69 ± 2.95 | 37.04 ± 0.88 | 37.63 ± 3.66 | 40.74 ± 0.79 |
| | 9.05 ± 0.31 | 19.68 ± 1.13 | 53.38 ± 5.90 | 148.16 ± 3.52 | 225.78 ± 21.96 | 325.92 ± 6.32 |
| **Methane** | 0 | 0 | 0 | 0.21 ± 0.20 | 1.10 ± 1.28 | 0.68 ± 0.04 |
| | 0 | 0 | 0 | 0.84 ± 0.80 | 6.60 ± 7.68 | 5.44 ± 0.32 |
| **Acetate anion** | 5.05 ± 0.57 | 5.5 ± 0.39 | 8.80 ± 0.35 | 14.71 ± 2.32 | 19.82 ± 1.37 | 18.86 ± 2.18 |
| | 2.53 ± 0.29 | 5.5 ± 0.39 | 17.60 ± 0.70 | 58.84 ± 9.28 | 118.92 ± 8.22 | 150.88 ± 17.44 |
| **Acetaldehyde** | 0.93 ± 0.01 | 0.78 ± 0.03 | 1.30 ± 0.37 | 0.92 ± 0.35 | 1.10 ± 0.06 | 0.91 ± 0.06 |
| | 0.47 ± 0.01 | 0.78 ± 0.03 | 2.60 ± 0.74 | 3.68 ± 1.40 | 6.60 ± 0.36 | 7.28 ± 0.48 |
| **Propionaldehyde** | 1.72 ± 0.04 | 1.14 ± 0.01 | 0.35 ± 0.30 | 0.05 ± 0.02 | 0.43 ± 0.01 | 0.53 ± 0.04 |
| | 0.86 ± 0.02 | 1.14 ± 0.01 | 0.70 ± 0.60 | 0.20 ± 0.08 | 2.58 ± 0.06 | 4.24 ± 0.32 |
| **Acetone** | 0.73 ± 0.17 | 0.38 ± 0.03 | 0.29 ± 0.06 | 0.26 ± 0.04 | 0.17 ± 0.03 | 0.25 ± 0.03 |
| | 0.37 ± 0.09 | 0.38 ± 0.03 | 0.58 ± 0.12 | 1.04 ± 0.16 | 1.02 ± 0.18 | 2.00 ± 0.24 |
| **Ethanol** | 9.78 ± 0.15 | 9.59 ± 0.53 | 12.61 ± 1.19 | 12.38 ± 1.00 | 8.28 ± 1.10 | 7.76 ± 0.45 |
| | 4.89 ± 0.08 | 9.59 ± 0.53 | 25.22 ± 2.38 | 49.52 ± 4.00 | 49.68 ± 6.60 | 62.08 ± 3.60 |
| **n-propanol** | 15.84 ± 0.72 | 14.53 ± 0.47 | 13.61 ± 0.49 | 6.63 ± 1.14 | 3.75 ± 0.59 | 3.13 ± 0.20 |
| | 7.92 ± 0.36 | 14.53 ± 0.47 | 27.22 ± 0.98 | 26.52 ± 4.56 | 22.50 ± 3.54 | 25.04 ± 1.60 |
| **Ally Alcohol** | 2.16 ± 0.05 | 2.66 ± 0.07 | 3.00 ± 0.10 | 1.81 ± 0.37 | 1.35 ± 0.13 | 0.94 ± 0.03 |
| | 1.08 ± 0.03 | 2.66 ± 0.07 | 6.00 ± 0.20 | 7.24 ± 1.48 | 8.10 ± 0.78 | 7.52 ± 0.24 |
| **1-butanol** | 0.07 ± 0.00 | 0.03 ± 0.01 | 0.02 ± 0.00 | 0.01 ± 0.00 | 0.01 ± 0.00 | 0.02 ± 0.00 |
| | 0.04 ± 0.00 | 0.03 ± 0.01 | 0.04 ± 0.00 | 0.04 ± 0.00 | 0.06 ± 0.00 | 0.16 ± 0.00 |
| **Methanol** | 0.24 ± 0.03 | 0.17 ± 0.01 | 0.19 ± 0.02 | 0.20 ± 0.01 | 0.17 ± 0.00 | 0.19 ± 0.01 |
| | 0.12 ± 0.02 | 0.17 ± 0.01 | 0.38 ± 0.04 | 0.80 ± 0.04 | 1.02 ± 0.00 | 1.52 ± 0.08 |
| **Oxygenates** | 36.51 ± 0.79 | 34.76 ± 1.47 | 40.18 ± 2.20 | 36.96 ± 0.98 | 35.07 ± 1.89 | 32.58 ± 1.56 |
| | 18.26 ± 0.40 | 34.76 ± 1.47 | 80.36 ± 4.40 | 147.84 ± 3.92 | 210.42 ± 11.34 | 260.64 ± 12.48 |

**Supplementary Table 13.** The CO-EC performance of the interface-rich sample at different applied current densities (unit: % for FE, mA $cm^{-2}$ for *j*).

| Products | 50 mA $cm^{-2}$<br>FE and *j* | 100 mA $cm^{-2}$<br>FE and *j* | 200 mA $cm^{-2}$<br>FE and *j* | 400 mA $cm^{-2}$<br>FE and *j* | 600 mA $cm^{-2}$<br>FE and *j* | 800 mA $cm^{-2}$<br>FE and *j* |
|---|---|---|---|---|---|---|
| **Hydrogen** | 53.87 ± 8.40<br>26.94 ± 4.20 | 51.16 ± 9.05<br>51.16 ± 9.05 | 41.19 ± 2.60<br>82.38 ± 5.20 | 43.20 ± 3.58<br>172.80 ± 14.32 | 22.47 ± 1.27<br>134.82 ± 7.62 | 20.48 ± 1.18<br>163.84 ± 9.44 |
| **Ethylene** | 18.36 ± 3.30<br>9.18 ± 1.65 | 19.03 ± 2.99<br>19.03 ± 2.99 | 25.30 ± 2.16<br>50.6 ± 4.32 | 21.27 ± 3.61<br>85.08 ± 14.44 | 37.46 ± 4.56<br>224.76 ± 27.36 | 36.12 ± 0.77<br>288.96 ± 6.16 |
| **Methane** | 0<br>0 | 0<br>0 | 0<br>0 | 0.83 ± 0.96<br>3.32 ± 3.84 | 0.12 ± 0.02<br>0.72 ± 0.12 | 0.26 ± 0.14<br>2.08 ± 1.12 |
| **Acetate anion** | 3.31 ± 0.47<br>1.66 ± 0.24 | 4.25 ± 0.39<br>4.25 ± 0.39 | 6.46 ± 0.52<br>12.92 ± 1.04 | 8.88 ± 0.88<br>35.52 ± 3.52 | 17.36 ± 4.65<br>104.16 ± 27.90 | 16.41 ± 1.63<br>131.28 ± 13.04 |
| **Acetaldehyde** | 1.06 ± 0.12<br>0.53 ± 0.06 | 0.92 ± 0.12<br>0.92 ± 0.12 | 0.99 ± 0.07<br>1.98 ± 0.14 | 0.80 ± 0.03<br>3.2 ± 0.12 | 0.53 ± 0.07<br>3.18 ± 0.42 | 1.08 ± 0.08<br>8.64 ± 0.64 |
| **Propionaldehyde** | 1.23 ± 0.35<br>0.62 ± 0.18 | 0.78 ± 0.37<br>0.78 ± 0.37 | 0.27 ± 0.09<br>0.54 ± 0.18 | 0.21 ± 0.07<br>0.84 ± 0.28 | 0.21 ± 0.15<br>1.26 ± 0.90 | 0.47 ± 0.33<br>3.76 ± 2.64 |
| **Acetone** | 0.14 ± 0.01<br>0.07 ± 0.01 | 0.13 ± 0.02<br>0.13 ± 0.02 | 0.21 ± 0.03<br>0.42 ± 0.06 | 0.25 ± 0.01<br>1.00 ± 0.04 | 0.16 ± 0.02<br>0.96 ± 0.12 | 0.39 ± 0.10<br>3.12 ± 0.80 |
| **Ethanol** | 11.88 ± 1.79<br>5.94 ± 0.90 | 13.43 ± 1.72<br>13.43 ± 1.72 | 15.21 ± 0.61<br>30.42 ± 1.22 | 19.10 ± 1.65<br>76.40 ± 6.60 | 12.39 ± 2.35<br>74.34 ± 14.10 | 14.57 ± 2.90<br>116.56 ± 23.20 |
| **n-propanol** | 9.09 ± 2.41<br>4.55 ± 1.21 | 8.49 ± 3.15<br>8.49 ± 3.15 | 7.53 ± 1.76<br>15.06 ± 3.52 | 4.84 ± 1.10<br>19.36 ± 4.40 | 6.16 ± 0.40<br>36.96 ± 2.40 | 6.40 ± 0.43<br>51.20 ± 3.44 |
| **Ally Alcohol** | 2.50 ± 0.20<br>1.25 ± 0.10 | 2.65 ± 0.46<br>2.65 ± 0.46 | 2.41 ± 0.11<br>4.82 ± 0.22 | 1.63 ± 0.49<br>6.52 ± 1.96 | 1.77 ± 0.04<br>10.62 ± 0.24 | 1.74 ± 0.17<br>13.92 ± 1.36 |
| **1-butanol** | 0.10 ± 0.02<br>0.05 ± 0.01 | 0.04 ± 0.01<br>0.04 ± 0.01 | 0.02 ± 0.02<br>0.04 ± 0.04 | 0.01 ± 0.01<br>0.04 ± 0.04 | 0<br>0 | 0<br>0 |
| **Methanol** | 0.57 ± 0.05<br>0.29 ± 0.03 | 0.62 ± 0.11<br>0.62 ± 0.11 | 0.59 ± 0.39<br>1.18 ± 0.78 | 0.92 ± 0.02<br>3.68 ± 0.08 | 0.23 ± 0.02<br>1.38 ± 0.12 | 0.33 ± 0.05<br>2.64 ± 0.40 |
| **Oxygenates** | 29.88 ± 5.26<br>14.94 ± 2.63 | 31.30 ± 5.34<br>31.30 ± 5.34 | 33.69 ± 0.42<br>67.38 ± 0.84 | 36.63 ± 1.17<br>146.52 ± 4.68 | 38.82 ± 6.61<br>232.92 ± 39.66 | 41.38 ± 1.68<br>331.04 ± 13.44 |

**Supplementary Table 14.** The Gibbs free energy change of the CO hydrogenation and CO dimerization reactions on different surfaces. The reference moleculars are CO and $H_2$ in the vacuum.

| | Ag(100) | Ag(111) | Cu(100) | Cu(111) |
|---|---|---|---|---|
| **$G_{ad,}$*HCO - $G_{ad,}$*COH / eV** | -0.67 | -0.86 | -0.67 | -0.13 |
| **$G_{ad,}$*HCO / eV** | 0.68 | 0.34 | -0.34 | -0.18 |
| **$\Delta G$ of CO hydrogenation / eV** | 0.45 | 0.47 | 0.56 | 0.69 |
| **$\Delta G$ of CO dimerization / eV** | 0.86 | 1.15 | 1.07 | 1.50 |

353

**Supplementary Note 1**

A techno-economic analysis (TEA) developed by Sargent et al.[3,4] is used to evaluate the economic feasibility of CO-to-oxygenate electroconversion. This method includes two parts: one is the costs for manufacturing, and the other is the total price of the produced products. Specifically, the costs for manufacture include 1. catalyst cost, 2. electricity cost, 3. separation cost, 4. feedstock cost, and 5. operation cost; the total prices of the produced products include hydrogen, ethylene, potassium acetate, n-propanol and ethanol (the other small fraction of oxygenates is omitted.).

1. The catalyst cost includes two parts: Cu and Ag. The market prices of Cu and Ag are USD 9286/ton and USD 757143/ton, respectively. The catalyst (Cu/30Ag) coverage is 1 $mg/cm^2$.
2. The electricity cost is calculated using USD 0.02 kW/h.
3. The separation cost includes the separation of unreacted feedstocks and the separation of products. The total separation cost is set as 30 % of the electricity cost.
4. Feedstock cost includes water and CO. The water and CO are USD 5/ton and USD 300/ton, respectively.
5. The operation cost is set as 10 % of (catalyst cost + separation device). The separation device is assumed to be 10 % of the catalyst cost.
6. The operational time of 80 % of a day (19.2 hours).
7. The electrosynthesis system is assumed to produce 80 tons/day potassium acetate. The conversion rate of CO is 55.1 %. The FEs for hydrogen, ethylene, potassium acetate, ethanol and n-propanol were 26.12 %, 13.29 %, 25.96 %, 26.04 %, 2.15 %. The cell potential and applied current density were 8 V and 800 $mA/cm^2$.
8. The market prices for hydrogen, ethylene, potassium acetate, ethanol and n-propanol are USD 1900, 1000, 1600, 800 and 1430/ton, respectively.

**Calculation content:**

(1) The total prices of the produced products include hydrogen, ethylene, potassium acetate, ethanol and n-propanol.

Yields of hydrogen, ethylene, potassium acetate, ethanol and n-propanol after the generation of 80 tons of potassium acetate:

$m_x = m_{potassium\ acetate}/M_{potassium\ acetate}$ * *electron transfer number for potassium acetate/*$(FE_{potassium\ acetate}/FE_x)$*/electron transfer number for x* * $M_x$;

(Here, x = hydrogen, ethylene, ethanol and n-propanol, corresponding to electron transfer numbers of 2, 8, 8 and 12, respectively.)

$m_{hydrogen}$ = (80 000 000/98*4/(25.96%/26.12 %)/2*2/1000 000) ton = 3.29 ton

$m_{ethylene}$ = (80 000 000/98*4/(25.96 %/13.29 %)/8*28/1000 000) ton = 5.85 ton

$m_{ethylene}$ = (80 000 000/98*4/(25.96 %/26.04 %)/8*46/1000 000) ton = 18.83 ton

$m_{n\text{-}propanol}$ = (80 000 000/98*4/(25.96 %/2.15 %)/12*60/1000 000) ton = 1.35 ton

*Thus, the total price of the produced products per ton of potassium acetate* = $\sum m_x/m_{potassium\ acetate}$*price of x per ton;*

(Here, x= hydrogen, ethylene, potassium acetate, ethanol and n-propanol)

the total price of the produced products per ton of potassium acetate = **1963.67** USD

(2) Electricity cost

*The charge used for cathodic electrolysis =* $[m_{acetate\ acid}/M_{acetate\ acid}$ ** electron transfer number ** $F/FE_{potassium\ acetate}]$ *C*

= (80 000 000/98 * 4 * 96485/25.96 %) C

= 1.21361E+12 C

*A 20 % loss is assumed during electrolysis; thus, the total charge is:*

[1.21361E+12/(1-20%)] C

= 1.51701E+12 C

*The electricity cost for one day = (The total charge/*$t_{one\ day}$ ** U/1000 ** $t_{one\ day}$ ** the price of kW* $h^{-2}$*) USD*

= (1.51701E+12/(19.2*3600) * 8/1000 * 19.2 * 0.02) USD

= 67422.76 USD

*The electricity cost for potassium acetate per ton = The electricity cost for one day/*$m_{potassium\ acetate}$

= (67422.76/80) USD

= **842.78** USD

(3) Feedstock cost

*Feedstock cost for CO = { [∑mx/Mx * (the carbon atom ratio of x per mole to CO per mole) ** $M_{CO}$*]/the conversion rate of CO * the price of CO per ton } USD;*

(Here, x= ethylene, potassium acetate, ethanol and n-propanol)

Thus, feedstock cost for CO = 44774.81.00 USD

*Feedstock cost for water = (Cathodic water for hydrogen evolution + anodic water for oxygen evolution) * water price per ton*

*=* $(m_{hydrogen}/M_{hydrogen}$ *** $M_{water}$ *+ the total charge/F/electron transfer number for oxygen evolution ** $M_{water})$ ** water price per ton*

Thus, the feedstock cost for water = 861.71 USD

*The feedstock cost for potassium acetate per ton = The feedstock cost of (CO + water)/*$m_{potassium\ acetate}$

= [(44774.81 + 861.71)/80] USD

= **570.46** USD

(4) Separation cost

*Separation cost = Electricity cost per ton * 30 %*

= (842.78 * 30 %) USD

= **252.84** USD

(5) Catalyst cost and separation device cost

*Catalyst cost = [(The total charge/$t_{one\ day}$/applied current density) * the catalyst cost per cubic meter] USD*

Thus, catalyst cost = 11578.78 USD

*Separation device cost = (Catalyst cost * 10 %) USD*

Thus, separation device cost = 1157.88 USD

*The total cost of catalyst and separation device for potassium acetate per ton = The cost of (catalyst + separation)/ $m_{potassium\ acetate}$*

= [(11578.78 + 1157.88)/80] USD

= **159.21** USD

(6) Operation cost

*Operation cost for potassium acetate per ton = (Catalyst cost + separation device cost) * 10 % USD*

= **15.92** USD

***In total, profit = (1) - (2) - (3) - (4) - (5) - (6) = 122.46 USD***

## Supplementary References

1. Nørskov, J. et al. Origin of the overpotential for oxygen reduction at a fuel-cell cathode. *J. Phys. Chem. B* **108**, 17886-17892 (2004).

2. Calle-Vallejo, F. & Koper, M. Theoretical considerations on the electroreduction of CO to $C_2$ Species on Cu(100) electrodes. *Angew. Chem. Int. Ed.* **52**, 7282-7285 (2013).

3. Leow, W. et al. Chloride-mediated selective electrosynthesis ofethylene and propylene oxides at highcurrent density. *Science* **368**, 1228-1233 (2020)

4. Lum, Y. *et al.* Tuning OH binding energy enables selective electrochemical oxidation of ethylene to ethylene glycol. *Nat. Catal.* **3,** 14-22 (2020).
