# Supplementary material for: High yield electrosynthesis of oxygenates from CO using a relay Cu-Ag co-catalyst system
Source: Nat Commun. 2024 May 8;15:3892. doi: 10.1038/s41467-024-48083-w (PMC11078980; doi:10.1038/s41467-024-48083-w)
Supplement: Supplementary file 3 — Description of Additional Supplementary Files [file 41467_2024_48083_MOESM3_ESM.pdf]

Description of Additional Supplementary Files

File Name: Supplementary Data 1
Description: The configuration files with all parameters for AIMD simulations and relaxed geometries through DFT are provided as Supplementary data 1.

File Name: Supplementary Video 1
Description: The transfer process of *HCO from Ag to Cu via AIMD simulations is provided in Supplementary Video 1.
